# Single-stranded DNA binding to the transcription factor PafBC triggers the mycobacterial DNA damage response

**DOI:** 10.1126/sciadv.adq9054

**Published:** 2025-02-07

**Authors:** Charlotte M. Schilling, Rafal Zdanowicz, Julius Rabl, Andreas U. Müller, Daniel Boehringer, Rudi Glockshuber, Eilika Weber-Ban

**Affiliations:** ^1^ ETH Zurich, Institute of Molecular Biology and Biophysics, 8093 Zurich, Switzerland.; ^2^ ETH Zurich, Cryo-EM Knowledge Hub, 8093 Zurich, Switzerland.

## Abstract

The DNA damage response in mycobacteria is controlled by the heterodimeric transcription factor PafBC, a member of the WYL domain–containing protein family. It has been shown that PafBC induces transcription of its regulon by reprogramming the housekeeping RNA polymerase holoenzyme to recognize PafBC-dependent promoters through sigma adaptation. However, the mechanism by which DNA damage is sensed and translated into PafBC activation has remained unclear. Here, we demonstrate that the binding of single-stranded DNA (ssDNA) to the WYL domains of PafBC activates the transcription factor. Our cryo–electron microscopy structure of full-length PafBC in its active conformation, bound to the transcription initiation complex, reveals a previously unknown mode of interaction between the ssDNA and the WYL domains. Using biochemical experiments, we show that short ssDNA fragments bind to PafBC dynamically, resulting in deactivation as ssDNA levels decrease postrepair. Our findings shed light on the mechanism linking DNA damage to PafBC activation and expand our understanding of WYL domain–containing proteins.

## Teaser

The structure of PafBC in its active conformation reveals the binding mode of the activating ligand at the PafBC WYL domains.

## INTRODUCTION

Mycobacteria, including the human pathogen *Mycobacterium tuberculosis* (Mtb), frequently encounter adverse conditions, many of which lead to DNA damage. To maintain their genome integrity, and thus ensure their survival, mycobacteria respond to DNA damage by inducing genes involved in DNA repair, recombination, and replication (*1*, *2*). Although some of these genes are regulated by the canonical LexA/RecA-dependent pathway known as the SOS response (*3*, *4*), most DNA damage response genes (~150) are controlled by the heterodimeric regulator PafBC (PafBC-dependent pathway) (*5*, *6*). PafBC acts as a transcriptional activator and belongs to the recently identified large bacterial family of WYL domain–containing proteins, which comprises predominantly transcription factors but also enzymes such as helicases (*7*–*9*). Although the WYL domain derived its name from a motif composed of a consecutive tryptophan-tyrosine-leucine sequence, this motif is only present in a subset of the members of this protein family. Instead, the common feature found in all WYL domains is a highly conserved, positively charged, ligand-binding groove that allows them to act as signal-transducing modules, which regulate the activities of the WYL domain–containing proteins (*7*, *8*). In addition to PafBC, several other WYL domain–containing transcription factors have recently been identified. This includes transcriptional repressors linked to bacterial immune systems (*10*–*12*), as well as two transcriptional activators: SiwR, a regulator of DinB/YfiT-like superfamily genes in *Mycobacterium smegmatis* (Msm) (*13*), and DriD, a DNA damage-inducible transcription factor that regulates *recA* and cell division inhibitor *didA* in *Caulobacter crescentus* (Ccr), a Gram-negative proteobacterium (*14*–*16*). Whereas the abovementioned transcription factors form homodimers (*10*–*13*, *15*), PafBC and its orthologs are the only heterodimeric WYL domain–containing proteins reported to date.

The first structural information on a WYL domain–containing protein was obtained with the crystal structure of *Arthrobacter aurescens* (Aau) PafBC in a DNA-free state (*17*). PafB and PafC each feature an N-terminal HTH domain, followed by the WYL domain and a WYL C-terminal extension (WCX) domain involved in heterodimer formation. The WYL domain exhibits an Sm-fold (*17*) commonly found in RNA binding proteins, such as the bacterial RNA chaperone Hfq (*18*, *19*). Previous studies indicated that PafBC levels remain constant even under conditions that damage DNA (*5*). This observation and the presence of the Sm-fold led to the hypothesis that the WYL domains of PafBC might function as a nucleic acid binding site transducing a stress signal, for example the presence of single-stranded DNA (ssDNA) during DNA damage, into a conformational change that activates PafBC (*17*). Corroborating this hypothesis, further experiments demonstrated an increase in the interaction between PafBC and RNA polymerase (RNAP) in the presence of ssDNA (*20*).

The mechanism of activation of the PafBC regulon genes was recently revealed by the cryo–electron microscopy (cryo-EM) structure of the PafBC-bound transcription initiation complex from *M. smegmatis* (*20*). This activation occurs under DNA-damaging conditions, where PafBC enables the housekeeping RNAP holoenzyme, associated with sigma factor A (SigA/σ^A^), to bind to PafBC-dependent promoters in a process called sigma adaptation. PafBC inserts itself in between the promoter DNA and domain 4 of SigA, the domain usually contacting the −35 motif of housekeeping promoters (*20*, *21*). In the PafBC-bound transcription initiation complex, the HTH domain of PafB engages with the PafBC-specific −26 promoter motif, whereas the HTH domain of PafC interacts with SigA domain 4 (*20*). Nevertheless, in this structure, it was not possible to resolve the domains of PafBC responsible for activation and to reveal the role of the DNA ligand in this process.

Here, using a real-time in vitro transcription assay, we demonstrate that PafBC is specifically activated through the binding of ssDNA. To elucidate the sequence of events leading to the formation of the RNAP-PafBC complex, we performed electrophoretic mobility shift assays (EMSAs). In addition, we present a cryo-EM structure of full-length PafBC in its active conformation, bound to the transcription initiation complex, revealing the presence of ssDNA extending across the PafB and PafC WYL domains. We show that the WYL domain of PafB is essential for ssDNA binding to PafBC and formation of the RNAP-PafBC complex. Our results provide key structural and mechanistic insights into the activation of PafBC in response to DNA damage in mycobacteria.

## RESULTS

### PafBC is activated by ssDNA binding at the WYL domains

**Fig. 1.**
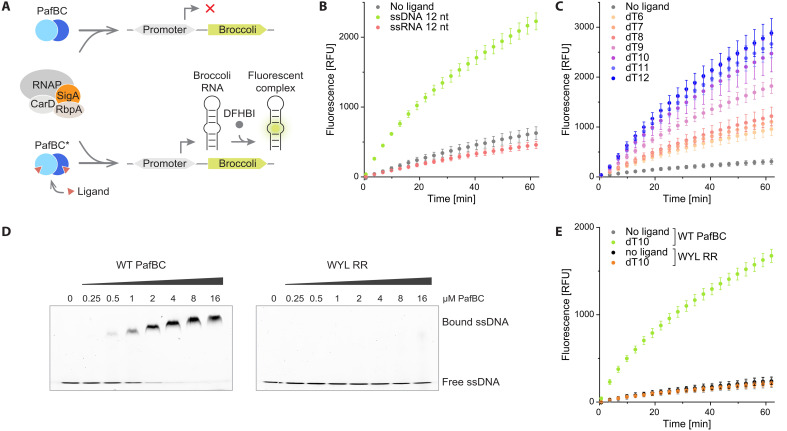
PafBC-mediated transcription is initiated by the binding of ssDNA to the PafBC WYL domains. (**A**) Scheme of the real-time in vitro transcription assay. Upon activation of PafBC, the RNA transcript Broccoli is produced and forms a fluorescent complex with the chromophore DFHBI. (**B**) Comparison of PafBC-dependent transcription observed over the course of 1 hour in the absence of ligand (gray) and upon addition of ssDNA (green; 5′-GTACAGTCGTAC-3′) or ssRNA (red; 5′-GUACAGUCGUAC-3′). RFU, relative fluorescence units. (**C**) Transcription assay using poly(dT) ssDNAs ranging from 6 to 12nt in length as PafBC activators. (**D**) EMSAs comparing the binding of WT PafBC and a PafBC variant (WYL RR) containing mutations of two arginines in each WYL domain (PafB: R211A/R214A and PafC: R201A/R204A) to 10 nM fluorescently labeled dT20 ssDNA. (**E**) Analysis of transcription levels upon dT10 ssDNA addition in the presence of either WT PafBC or WYL RR.

PafBC-dependent transcription activation requires the association of PafBC with the RNAP-σ^A^ holoenzyme that is bound to double-stranded DNA (dsDNA) containing the PafBC-specific promoter motif. In a pull-down experiment with Strep-tagged RNAP, the amount of PafBC retained with RNAP was shown to be substantially increased when a 12–nucleotide (nt)-long ssDNA molecule was added to the reaction, suggesting that PafBC is activated by ssDNA binding (*20*). To explore which forms of ssDNA can serve as stress-transducing ligands and to assess whether they activate PafBC to varying extents, we implemented an in vitro transcription assay to monitor transcription in real-time using the *M. smegmatis* transcription machinery. The readout is based on an RNA aptamer called “Broccoli” that forms a fluorescent complex with 3,5-difluoro-4-hydroxybenzylideneimidazolinone (DFHBI), a synthetic molecule designed to mimic the chromophore of green fluorescent protein (GFP) (*22*, *23*). The transcription reaction is carried out with a dsDNA template carrying the required promoter and encoding the RNA aptamer able to form the fluorescent complex with DFHBI (Fig. 1A). The promoter template was designed based on the promoter region of the *recA* gene, which contains both a PafBC-dependent promoter (P1) and a PafBC-independent promoter (P2) controlled by LexA/RecA. Because the LexA/RecA-dependent promoter is irrelevant for PafBC-dependent transcription, it was removed to create the PafBC-dependent transcription template used in this study. For the control template, both promoter regions were deleted (fig. S1A). In addition to RNAP and housekeeping sigma factor A, actinobacteria-specific RNAP binding protein A (RbpA) and CarD were also added to the in vitro transcription reactions (Fig. 1A).

Without the addition of a ligand, DFHBI fluorescence increased slightly over time (Fig. 1B). This is likely due to nonspecific binding of RNAP-σ^A^ to the linear promoter DNA and sporadic, unspecific initiation of transcription. A similar basal level of transcription was observed in a recent study on an *M. tuberculosis* in vitro transcription assay using a different RNA aptamer (*24*). In the presence of the 12-nt-long ssDNA used in the pull-down experiment mentioned above, fluorescence increased considerably over time, reporting on substantial RNA production (Fig. 1B). This increase in fluorescence is strictly PafBC-dependent, as the addition of ssDNA in the absence of PafBC led to fluorescence levels comparable to the control experiment without the ssDNA ligand (fig. S1B). Similarly, when ssDNA was added to a reaction containing a DNA template lacking the PafBC-dependent promoter, fluorescence did not exceed the level obtained without a ligand (fig. S1C). These results support a mechanism of activation where the binding of ssDNA leads to enhanced complex formation between PafBC and RNAP-σ^A^ and consequently to specific transcription initiation at PafBC-dependent promoters.

Some proteins featuring an Sm-fold, such as RNA chaperone Hfq or WYL1, an accessory protein of the Type VI-D CRISPR-Cas system, were shown to bind single-stranded RNA (ssRNA) (*18*, *25*). Therefore, we next tested a 12-nt-long ssRNA molecule with its sequence equivalent to that of the ssDNA described above. However, the addition of ssRNA did not result in PafBC activation (Fig. 1B). In addition, the effects of various nucleotide second messengers including cAMP [3′,5′-cyclic adenosine monophosphate (AMP)], cGAMP [2′,3′- and 3′,3′-cyclic guanosine monophosphate (GMP)–AMP], and pppGpp (guanosine-3′,5′-pentaphosphate) were evaluated. However, none exhibited transcriptional activity beyond the control level observed in the absence of a ligand (fig. S2A). When comparing RNA production upon addition of 10-nt-long ssDNA molecules containing only one type of base, we found poly-thymidine (dT) ssDNA to activate PafBC most efficiently (fig. S2B). We then tested poly(dT) ssDNAs between 6 and 12 nt in length to explore the impact of the oligonucleotide length on transcription activation. We found that longer ssDNAs were more efficient in activating PafBC (Fig. 1C), although ssDNA as short as 6 nt already resulted in a considerable increase in transcription. ssDNA ligands (added in excess relative to the dsDNA template) longer than 12 nt could not be tested in the transcription assay as this abolished transcription, most likely due to nonspecific binding of RNAP-σ^A^ to the ssDNA.

The WYL domains were previously hypothesized to function as ligand-binding modules, owing to their structural similarity to the RNA chaperone Hfq (*17*). Supporting this hypothesis, mutation of two highly conserved arginine residues in the PafB and PafC WYL domains was shown to result in decreased viability of *M. smegmatis* under DNA-damaging conditions (*17*). Using EMSAs, we demonstrate that wild-type (WT) PafBC binds ssDNA with low micromolar affinity (Fig. 1D). In contrast, a PafBC variant (WYL RR), in which the two highly conserved arginine residues in both WYL domains were mutated to alanines (PafB: R211A/R214A and PafC: R201A/R204A), could no longer bind to ssDNA (Fig. 1D). Consistently, no transcriptional activation was observed following ssDNA addition when the PafBC WYL RR variant was used (Fig. 1E). These results establish that the binding of ssDNA to the WYL domains is the DNA damage-sensing step that renders PafBC active.

### Binding of PafBC to RNAP-σ^A^ requires prior association of RNAP-σ^A^ with PafBC-specific promoter DNA

To investigate the sequence of events during formation of the RNAP-PafBC transcription initiation complex, we studied the binding of PafBC to dsDNA as well as to RNAP-σ^A^ using EMSAs. Mycobacterial RNAP-σ^A^ open complexes, in which DNA strands are separated within the transcription bubble, are known to form reversibly and transiently (*26*). To stabilize the open complex, we introduced a noncomplementary transcription bubble at the −10 region of all dsDNA templates [as described in (*20*)].

**Fig. 2.**
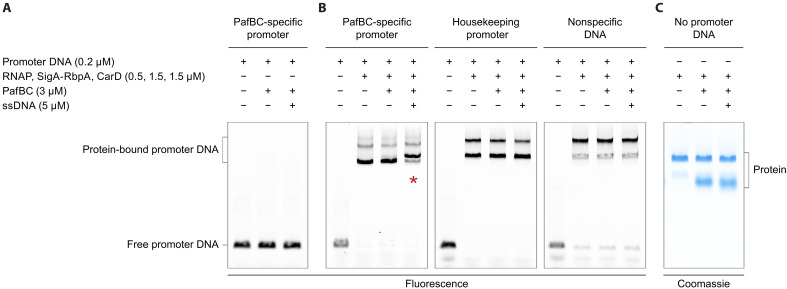
The RNAP-PafBC complex forms only with RNAP-σ^A^ already bound to PafBC-specific promoter DNA. (**A**) PafBC binding to PafBC-specific promoter DNA was analyzed using EMSAs. PafBC was added to fluorescently labeled promoter DNA in the presence or absence of dT12 ssDNA. Because the *M. smegmatis* open complex has been shown to be unstable against dissociation, all DNA scaffolds used in this experiment contained a preformed transcription bubble at the −10 region to increase complex stability. (**B**) RNAP-PafBC complex formation was studied by EMSAs with fluorescently labeled promoter DNA containing either the PafBC-specific, the housekeeping, or no promoter motif. RNAP holoenzyme was added either alone, with PafBC, or with PafBC and dT12 ssDNA. A shift of the RNAP-σ^A^ band, indicative of RNAP-PafBC complex formation, was observed only when PafBC and ssDNA were added to the PafBC-specific promoter dsDNA (red asterisk). (**C**) RNAP-PafBC complex formation in the absence of a promoter dsDNA template was examined by native PAGE and Coomassie staining. PafBC and ssDNA addition did not result in a shift of the RNAP-σ^A^ band, showing that the RNAP-PafBC complex was not formed.

Our results show that PafBC, in the absence of RNAP holoenzyme, does not interact with dsDNA containing the PafBC-specific promoter motif, regardless of the presence or absence of an ssDNA ligand, as no mobility shift of the labeled dsDNA probe was observed (Fig. 2A). This observation was independent of whether a mismatch at the −10 region of the dsDNA template was present or not (fig. S2C). In contrast, RNAP-σ^A^ bound nonspecifically to dsDNA, as evidenced by the mobility shift of the single band seen for free dsDNA template into two main higher mass bands containing RNAP holoenzyme. This pattern was consistent for all dsDNA templates tested: a PafBC-specific promoter template, a housekeeping promoter template containing a −35 and a −10 motif, and a promoterless template of similar length (Fig. 2B).

Notably, in the absence of ssDNA, the addition of PafBC to RNAP-σ^A^ bound to any one of the dsDNA templates did not result in the formation of the RNAP-PafBC complex. However, a mobility shift indicative of RNAP-PafBC complex formation was observed when ssDNA was also added, and this occurred only with RNAP-σ^A^ bound to PafBC-specific promoter DNA. Supporting this finding, native polyacrylamide gel electrophoresis (PAGE) analysis revealed that the RNAP-PafBC complex does not form when RNAP-σ^A^ is not bound to a promoter template, regardless of ssDNA presence (Fig. 2C). Together, these results suggest that PafBC is first activated by ssDNA before it associates with RNAP-σ^A^ that must already be bound to PafBC-specific promoter DNA.

### Cryo-EM structure of PafBC in its active conformation

With the aim to obtain a structure of the active transcription initiation complex where the full-length structure of PafBC could be resolved, we used formaldehyde cross-linking prior to purifying of the RNAP-PafBC complex by size exclusion chromatography (fig. S3). Initially, we collected cryo-EM data from the PafBC-containing transcription initiation complex assembled in the presence of CarD, a global transcriptional regulator essential in mycobacteria (*27*, *28*). In our previously published study CarD was present in the cryo-EM sample but was not detected in the structure of the PafBC-bound transcription initiation complex. In the current study, three-dimensional (3D) class averages of the RNAP-PafBC complex with and without CarD were identified during data processing, demonstrating that PafBC and CarD can simultaneously bind to the RNAP holoenzyme (fig. S4, A and B). Comparison of in vitro transcription levels in the presence and absence of CarD revealed that CarD substantially increased PafBC-mediated transcription (fig. S4C), underscoring its role in stabilizing the transcription bubble formed by the PafBC-dependent open complex. However, EMSA experiments showed no effect of CarD on the formation of the RNAP-PafBC complex (fig. S4D). This apparent discrepancy likely arises from the preformed transcription bubble in the promoter dsDNA used in both the EMSAs and for cryo-EM sample preparation, as it is expected to reduce the requirement for CarD to stabilize the open complex. Therefore, to minimize particle heterogeneity, CarD was excluded from the next cryo-EM sample.

**Fig. 3.**
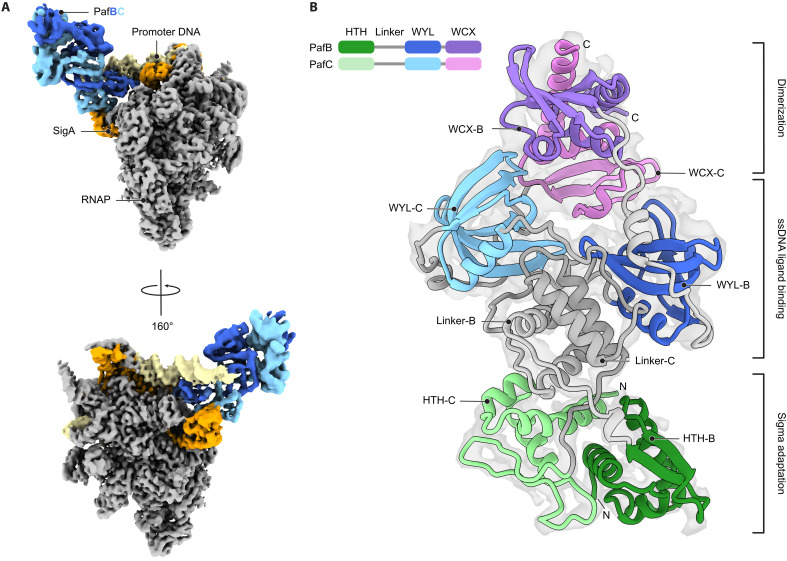
Structure of PafBC in its active conformation bound to the transcription initiation complex. (**A**) Separately refined maps of PafBC (map_PafBC_: blue) and RNAP complex (map_RNAP_: promoter DNA, pale yellow; SigA, orange; RNAP subunits and RbpA, gray) were fitted to the RNAP-PafBC composite map of the whole complex. (**B**) Structural model of PafBC refined against map_PafBC_. The domains are colored according to the schematic diagram of the domain organization displayed on the top left.

Cryo-EM single-particle analysis of the RNAP-PafBC transcription initiation complex, assembled without CarD, yielded a structure of full-length PafBC. The complex included RNAP, SigA, RbpA, and PafBC from *M. smegmatis*, along with dsDNA containing a PafBC-dependent promoter with a preformed transcription bubble, and an 11-nt-long ssDNA. Because of considerable conformational heterogeneity of PafBC relative to the RNAP holoenzyme, the two components were refined separately to global resolutions of 4.0 Å for PafBC and 3.3 Å for RNAP holoenzyme (figs. S5 and S6). These two 3D reconstructions were then combined to generate the composite map of the fully assembled RNAP-PafBC complex (Fig. 3A).

Within the transcription complex, PafBC adopts an elongated, pseudo-twofold symmetric structure, with PafB and PafC exhibiting similar but nonidentical conformations (Fig. 3B and fig. S7, A and B). The HTH domains are arranged as described previously (*20*) and are connected to the WYL domains through long linkers. Each linker contributes two helices to form a four-helix bundle (fig. S7C). This configuration contrasts with the linkers observed in the ^Aau^PafBC crystal structure, where the PafB linker is a single, extended α helix and the PafC linker forms a three-helix bundle (fig. S7C) (*17*). In our structure, the WYL domains are positioned diametrically, forming the broadest part of the heterodimer. They connect to the C-terminal WCX domains via flexible linkers providing additional flexibility to the WCX domains. The ferredoxin-like fold of the two WCX domains from ^Msm^PafBC reported here, as well as their interaction with each other, is structurally similar (root mean square deviation = 2.04 Å) to the WCX domains of ^Aau^PafBC solved by x-ray crystallography (*17*).

### PafBC forms a tunnel for ssDNA binding

**Fig. 4.**
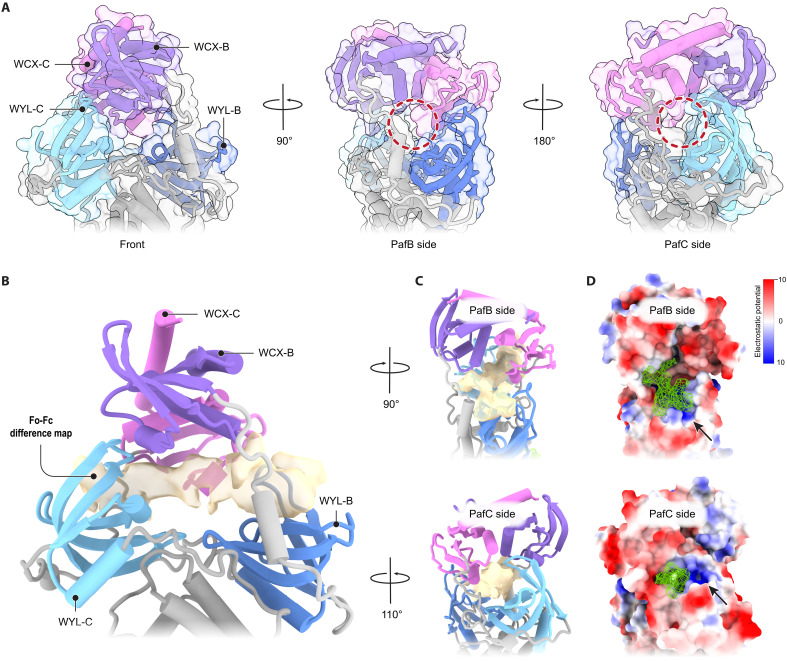
ssDNA is bound at the PafBC WYL domains. (**A**) Close-up view of the WYL-WCX region of PafBC. Openings into a tunnel piercing across the complex are highlighted on the side of PafB and PafC, respectively (red circle). (**B**) Fo-Fc difference density map (beige, contour level 21.9, dust hidden) within the PafBC model reveals continuous density at the interface between the WYL and WCX domains connecting the PafB and PafC WYL domains. (**C**) Side views of the PafBC model and the Fo-Fc difference density map (beige). (**D**) Surface representation of the PafBC model colored by electrostatic potential showing the Fo-Fc difference density as mesh (green). The clustering of positively charged residues is visible at both the PafB and PafC openings into the tunnel (indicated by black arrows). The electrostatic potential key is shown on the top right.

**Fig. 5.**
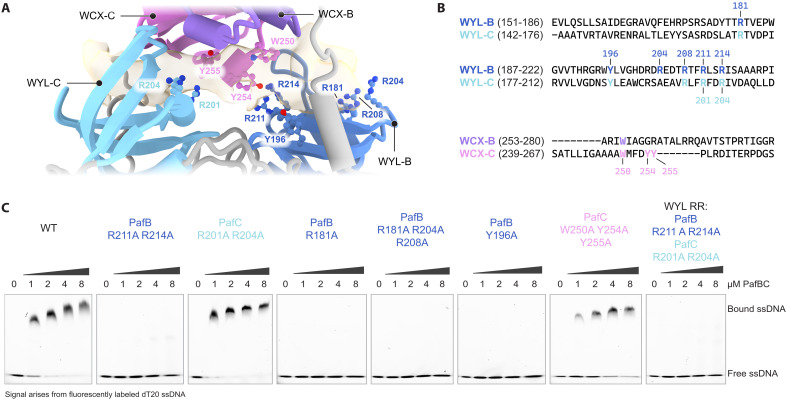
Mutations in the PafB WYL domain result in loss of ssDNA binding. (**A**) Close-up view of the WYL-WCX region of the PafBC model displaying the Fo-Fc difference density map (beige, contour level 21.9, dust hidden). Residues potentially interacting with the ssDNA are labeled. WYL-B and WYL-C are colored in dark and light blue, and WCX-B and WCX-C are colored in dark and light purple, respectively. (**B**) Sequence alignment of the WYL and part of the WCX domains from *M. smegmatis* PafB and PafC. Residues shown in (A) are colored according to their corresponding domain. (**C**) ssDNA binding to WT PafBC and PafBC variants containing mutations in the WYL or WCX domains was analyzed using EMSAs. WT PafBC and PafBC variants were titrated against 10 nM fluorescently labeled dT20 ssDNA.

In our RNAP-bound structure of PafBC, the previously proposed ligand-binding grooves of the WYL domains are oriented toward the WCX domains. In the surface representation of the refined PafBC model, a distinct tunnel is visible between the WYL and WCX domains, extending across the entire heterodimer (Fig. 4A). The Fo-Fc difference density map revealed extra density within this tunnel, linking the WYL domains of PafB and PafC (Fig. 4, B to D). The abovementioned arginine residues (PafB: R211/R214 and PafC: R201/R204), which are essential for ssDNA binding to PafBC, are oriented toward this additional density (Fig. 5, A and B), substantiating its identity as the ssDNA ligand. These observations demonstrate that our structure captured PafBC in an active state, complexed with ssDNA.

The surface representation colored by the electrostatic potential reveals that the ligand-binding sites of both PafB and PafC are strongly positively charged due to the presence of several arginine residues (Fig. 4D). Although the overall fold of PafB and PafC is similar, the distance between the WYL and WCX domains in PafB is considerably larger than the corresponding distance in PafC. As a result, the positively charged groups are more exposed on the side of PafB (Fig. 4, C and D). We hypothesized that this positional difference between the PafB and PafC WYL domains could correspond to a functional difference. To investigate this theory, we mutated the two highly conserved arginine residues that are present in both WYL domains in either only the PafB or only the PafC WYL domain (PafB WYL: R211A/R214A and PafC WYL: R201A/R204A; fig. S8). Using EMSAs to assess ssDNA binding, we observed that the PafC WYL variant bound ssDNA with an affinity comparable to WT PafBC. In contrast, ssDNA binding was completely abolished in the variant carrying the PafB WYL mutation (Fig. 5C). Consistent with this finding, the PafB WYL variant was also no longer able to bind to RNAP-σ^A^ in the presence of ssDNA, whereas the PafC WYL variant formed the RNAP-PafBC complex with similar efficiency as WT PafBC (fig. S9).

We further explored the role of the PafB WYL domain by mutating additional highly conserved residues, including three arginine residues (R181, R204, and R208) that form a positively charged patch at the entrance of the PafB ssDNA binding groove, as well as tyrosine Y196 that is part of the WYL motif (Fig. 5, A and B, and fig. S8). All PafBC variants with mutations in the PafB WYL domain (R181A or R181A/R204A/R208A or Y196A) exhibited a complete loss of ssDNA binding (Fig. 5C). Although the Y196A retained the ability to associate with the RNAP holoenzyme, the R181A variant showed substantially less RNAP-PafBC complex formation compared to WT PafBC (fig. S9). Moreover, the triple arginine variant (R181A/R204A/R208A) was entirely incapable of forming a complex with RNAP-σ^A^ (fig. S9). These observations underscore the critical role of the PafB WYL domain in ssDNA binding and transcription activation.

**Fig. 6.**
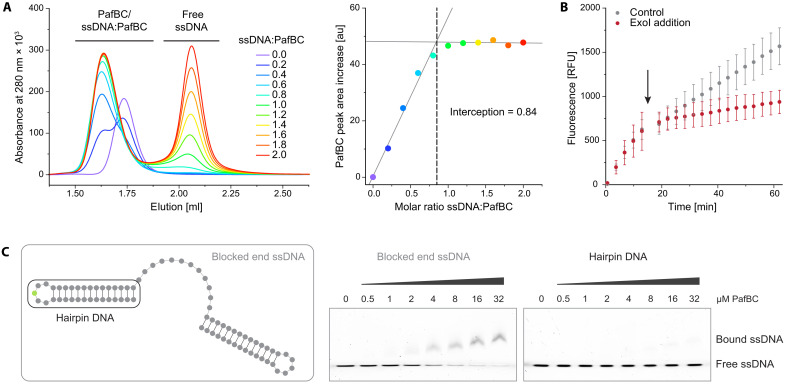
PafBC binds dynamically to a single ssDNA molecule and can associate with different forms of ssDNA. (**A**) Titration of PafBC with dT20 ssDNA, analyzed by analytical gel filtration chromatography (left). The peak areas corresponding to the sum of PafBC and ssDNA:PafBC complex were determined. The peak area increase over the PafBC peak in the absence of ssDNA (purple curve) represents the amount of ssDNA in the complex since the PafBC concentration remains constant. The PafBC peak area increase was plotted against the molar ratio of ssDNA:PafBC (right). au, arbitrary units. (**B**) Comparison of PafBC-dependent transcription in the presence of ssDNA (5′-GTACAGTCGTAC-3′) upon addition of either ExoI (red) or ExoI storage buffer (control; gray) after 15 min (indicated by an arrow). (**C**) The binding of PafBC to 10 nM of a fluorescently labeled (FAM-dT indicated in green) DNA construct containing a dT12 ssDNA stretch flanked by two hairpin loops (blocked end ssDNA, indicated by the gray box on the left) was analyzed by EMSA (middle). The binding of PafBC to the 10 nM control DNA construct comprising just the hairpin DNA (indicated by the black box on the left) is shown on the right.

Intriguingly, our cryo-EM structure revealed that the ssDNA ligand contacts not only the PafB but also the PafC WYL domain. Previous in vivo analysis showed that mutations in either WYL domain result in a viability defect under DNA-damaging conditions (*17*). To complement our structural findings, we performed analytical gel filtration experiments in which PafBC was titrated with ssDNA. This yielded an ssDNA-to-PafBC stoichiometry of ~1:1, consistent with our cryo-EM model (Fig. 6A). Furthermore, we tested a PafBC variant containing substitutions of three aromatic residues, located at the center of the ssDNA binding tunnel (W250A/Y254Y/Y255A). On the basis of their proximity to the ssDNA density in the structure, we hypothesized that these conserved residues might contribute to ssDNA binding (Fig. 5, A and B, and fig. S8). Our experiments showed that this PafC WCX variant retained the ability to bind both ssDNA and RNAP holoenzyme, albeit to a lesser extent (Fig. 5C and fig. S9). These results provide additional evidence that the ssDNA traverses through the binding tunnel.

### PafBC can bind to ssDNA gaps

We assumed that, in the physiological context, ssDNA binding to PafBC primarily depends on ssDNA concentration. To address this experimentally, we performed two in vitro transcription reactions, both containing PafBC and an ssDNA ligand. After 15 min, Exonuclease I (ExoI), an ssDNA-specific exonuclease, was added to one reaction, whereas the other received only ExoI storage buffer as a control. Transcription levels were then further monitored over time. In the control reaction, fluorescence continued to increase as expected. In contrast, transcription decreased sharply in the ExoI-treated reaction (Fig. 6B). These results suggest that ssDNA dissociation from PafBC occurs independently of additional factors, supporting our model that ssDNA concentration regulates PafBC activity.

The structural analysis revealed that ssDNA binds within a topologically enclosed binding tunnel in PafBC, raising the question of whether PafBC requires free ssDNA ends for binding and what type of ssDNA sources might serve to activate PafBC in vivo. To address this, we performed EMSAs using a fluorescently labeled DNA construct containing a dT12 ssDNA segment flanked by two hairpin loops (Fig. 6C). PafBC was able to bind this construct, whereas it failed to interact with a control construct comprising only the DNA hairpin. This demonstrates that a free ssDNA end is not essential for PafBC binding. Collectively, our results support a model in which ssDNA initially associates with the PafB WYL domain triggering a conformational change in PafBC. This conformational change facilitates the formation of the ssDNA-PafBC complex, where the ssDNA is positioned within an internal tunnel that is lined with positively charged and aromatic residues and that connects the WYL domains of PafB and PafC.

## DISCUSSION

The mycobacterial DNA damage response is activated by the heterodimeric transcription factor PafBC, a member of the large family of WYL domain–containing proteins found almost exclusively in bacteria (*5*, *6*, *17*). Recent studies revealed that PafBC activates transcription of its regulon genes by reprogramming the canonical RNAP holoenzyme to recognize PafBC-dependent promoters in a process called sigma adaptation (*20*). However, it has remained unclear by which mechanism DNA damage results in PafBC activation. In this study, we presented structural and biochemical evidence for the specific activation of PafBC by binding of an ssDNA ligand to its WYL domains via an ssDNA binding tunnel extending across the widest part of the heterodimer.

The presence of ssDNA is a hallmark of DNA damage in both eukaryotes and prokaryotes as it is frequently produced at DNA lesion sites, e.g., by DNA end resection at double-strand breaks or stalling of replication forks (*29*, *30*). The canonical DNA damage response pathway of bacteria, the SOS response, is activated by RecA filaments, which form around ssDNA sites and promote the autocatalytic cleavage of the repressor LexA (*3*, *4*). Using a real-time in vitro transcription assay, we monitored the production of RNA transcript upon addition of either ssDNA or ssRNA and found that PafBC is specifically activated by ssDNA. Because PafBC does not require a free ssDNA end for binding, we hypothesize that PafBC can be activated in vivo by various sources of ssDNA, including ssDNA tails generated by DNA end resection, short free ssDNA fragments, and ssDNA gaps. Nucleotide excision repair, for example, produces ssDNA fragments of 12- to 13-nt in length in mycobacteria (*31*). Although the stability against degradation of such short fragments is not well understood, PafBC could potentially use them for activation. In addition, ssDNA gaps at stalled replication forks as well as R-loops, which are composed of a DNA-RNA hybrid and an exposed ssDNA strand, arise more frequently during DNA damage and might be able to trigger PafBC activation as well. However, movement of genome-associated forms of ssDNA over long distances to PafBC-dependent promoters seems unlikely. It is more plausible that such sources of ssDNA would have to be more local.

The activation of both RecA and PafBC by ssDNA prompts the question of why mycobacteria and other actinobacteria have evolved two distinct DNA damage repair pathways, regulated by the same type of ligand. We showed that longer ssDNA molecules lead to more effective PafBC activation, although ssDNA as short as 6 nt can still induce some activation. This suggests that PafBC can respond to comparatively short ssDNA fragments that RecA cannot use for nucleation of protofilaments. Moreover, differences in activation between RecA and PafBC may arise from DNA modifications or the involvement of other proteins, despite both being triggered by a similar ligand (*32*). Given the structural flexibility of PafBC and the spacious central tunnel through which the ssDNA passes, we speculate that DNA modifications such as alkylation, oxidation, or cyclobutene pyrimidine dimers could be accommodated.

Previous research has shown that, although PafBC regulates most DNA damage repair genes in mycobacteria (*6*), both the SOS response and the PafBC-dependent pathway are required for comprehensive DNA damage repair under diverse conditions (*33*). Specifically, the SOS response proved important for addressing UV radiation damage, whereas PafBC is crucial for survival following gyrase inhibition (*33*). These differences may partly reflect differences in the types of ssDNA generated by various DNA-damaging agents. Furthermore, the evolution of two DNA damage repair pathways may enhance the robustness of the DNA damage response, providing greater temporal regulation and adaptability depending on the nature and severity of the damage (*34*). Future research into the potential differences in ssDNA ligands and the conceivable competition between PafBC and RecA for ssDNA will be crucial for elucidating the interplay between these two mycobacterial DNA damage response pathways.

Our cryo-EM map of full-length PafBC bound to the transcription initiation complex revealed the density for the activator ssDNA bridging the two WYL domains, a finding supported by structure-based mutagenesis experiments performed in this study. These results also provide context for prior observations of in vivo viability experiments, where the survival of *M. smegmatis* ∆*pafBC* strains complemented with either WT PafBC or different PafBC variants was compared in the presence of the DNA-damaging agent mitomycin C (*17*). Mutations in both WYL domains (PafB: R211A/R214A and PafC: R201A/R204A) lowered viability to levels comparable to the knockout strain, whereas mutations in either the PafB (R211A/R214A) or the PafC (R201A/R204A) WYL domain resulted in moderately reduced viability. Although the lower resolution of the ssDNA density precludes structural modeling, we show that, instead of two ssDNA molecules binding to the two PafBC WYL domains, a single ssDNA molecule extends across the heterodimer and contacts both WYL domains simultaneously. This agrees with the results of our in vitro transcription assay, where we found that longer ssDNA molecules activate PafBC more efficiently. Considering the reported length of 6.8 Å per ssDNA nucleotide (*35*), the calculated minimum length of ssDNA necessary to connect both WYL domains (PafB R204 to PafC R204) is 6 nt. The observation of ssDNA density beyond PafC R204, however, explains why longer ssDNA molecules activate PafBC more strongly. Although both WYL domains interact with the ssDNA ligand, we found that mutations in the PafB WYL domain disrupt ssDNA binding and impede RNAP-PafBC complex formation, whereas mutations in the PafC WYL domain lead to a behavior similar to WT PafBC in these assays. These findings suggest that the ssDNA ligand first engages with the PafB WYL domain.

**Fig. 7.**
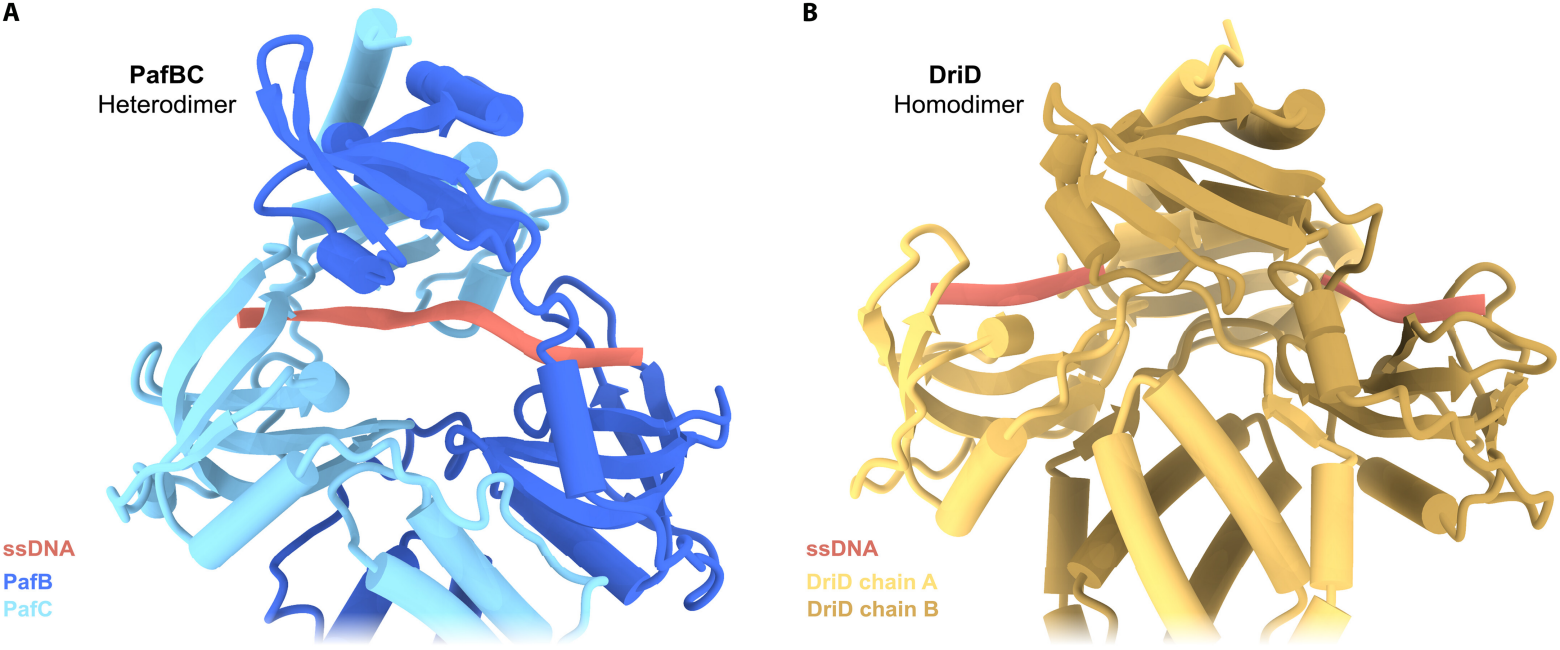
Comparison of ssDNA binding in PafBC and DriD. (**A**) *M. smegmatis* PafBC structure as part of the RNAP transcription initiation complex with ssDNA (salmon) modeled within the Fo-Fc difference density map for representative purposes. PafB and PafC are colored in shades of blue. (**B**) Crystal structure of the homodimeric transcriptional regulator DriD from *C. crescentus* (PDB: 8TP8). Each WYL domain is occupied with one ssDNA molecule.

The mode of ssDNA binding in PafBC differs from the only other ssDNA-WYL domain interaction reported so far. A recent crystal structure of DriD, a homodimeric DNA damage-inducible transcription factor that triggers the SOS-independent transcription of a small regulon in *C. crescentus*, was solved in complex with promoter dsDNA and ssDNA ligands (*16*). This structure revealed two ssDNA molecules associated with DriD, one per WYL domain, which do not penetrate the protein in the same manner as seen in PafBC (Fig. 7, A and B). We speculate that this difference in binding mode might be linked to the heterodimeric nature of PafBC. The asymmetric orientation of its HTH domains relative to the pseudo-twofold symmetry axis is essential for interaction with the RNAP holoenzyme. It is likely that the observed differences in ssDNA binding between the WYL domains of PafB and PafC promote this asymmetry. Furthermore, different from PafBC, several WYL domain–containing transcription factors have been shown to bind to their respective promoter dsDNAs in the absence of ligands. This includes DriD [dissociation constant(*K*_D_) of 25 nM]; the *M. smegmatis* DinS operon regulator SiwR (*K*_D_ of 651 nM); the CBASS-associated transcription factor CapW from *Escherichia coli* UPEC-117, *Stenotrophomonas maltophilia* C11, and *Pseudomonas aeruginosa* (*K*_D_ of 0.3, 1.5, and 1.0 μM, respectively); the anthraquinone expression regulator AntJ from *Photorhabdus luminescens* (*K*_D_ of 100 nM); and the BREX system repressor BrxR from *Acinetobacter* (*10*, *12*, *13*, *15*, *36*). Consistently, a recently determined structure of BrxR shows that both HTH domains interact with the promoter dsDNA (*10*), analogous to DriD (*16*). Collectively, these findings imply that, although all the abovementioned WYL domain–containing proteins are involved in transcription regulation, the regulatory mechanisms likely differ depending on the homo- or heterodimeric nature of the regulator and the type of ligand binding at the WYL domains.

A recently reported structure of a *Streptomyces coelicolor* transcription initiation complex involving AfsR, a Streptomyces antibiotic regulatory protein, demonstrated that AfsR operates through a mechanism resembling sigma adaptation, enabling initiation from imperfect −35 regions (*37*). However, unlike PafBC, AfsR lacks WYL domains and is activated through a different pathway. Moreover, the mechanism of sigma adaptation by AfsR differs from that of PafBC, and AfsR establishes additional contacts with the C-terminal domains of the RNAP α subunits. Despite these differences, this finding supports the hypothesis that transcriptional activation by sigma adaptation is a more widespread phenomenon in actinobacteria that can be triggered by different signals.

The structure of PafBC presented here differs from the previously reported ^Aau^PafBC structure obtained by x-ray crystallography (*17*). In the current structure, both HTH domains are accessible and interact with either the promoter dsDNA or the RNAP holoenzyme. In contrast, the ^Aau^PafBC structure solved without an ssDNA ligand shows HTH domain positioning that precludes simultaneous interaction with both the RNAP-σ^A^ and the promoter DNA. The linker region likely plays an important role in transducing the signal of ssDNA binding, as the bound ssDNA stabilizes the linker in a specific conformation, potentially by constraining the distance between the two WYL domains. This, in turn, stabilizes PafBC in its active, pseudo-twofold symmetric conformation. It is unclear whether the crystal structure represents “the” inactive conformation of PafBC or merely one of several possible inactive conformations. Future studies exploring the flexibility of PafBC in the absence and presence of ssDNA, along with time-resolved analyses of its mechanism of activation, will be critical for unraveling the conformational dynamics of PafBC.

**Fig. 8.**
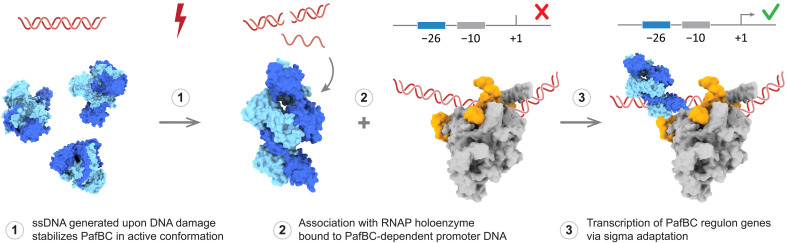
Proposed model of PafBC-dependent sigma adaptation.

Our biochemical experiments exploring the order of binding events during PafBC-dependent transcription initiation revealed that PafBC does not bind to PafBC-promoter dsDNA in the absence of RNAP holoenzyme, irrespective of the presence of ssDNA ligand. In contrast, RNAP-σ^A^ associated promiscuously with all dsDNA templates tested. Although the precise mechanism by which RNAP-σ^A^ searches for promoter motifs has not been fully elucidated, nonspecific interactions of RNAP-σ^A^ with DNA were shown to facilitate the promoter search (*38*–*40*). PafBC formed a complex exclusively with RNAP-σ^A^ bound to PafBC-specific promoter DNA and only in the presence of the ssDNA ligand. Without dsDNA, the RNAP-PafBC complex was not formed. Combined, these results support a model in which RNAP-σ^A^ scans along the DNA, halts at the PafBC promoter, and subsequently binds to PafBC that has been activated by ssDNA binding (Fig. 8).

Combined, our findings reveal that ssDNA binding at the WYL domains allosterically activates PafBC, elucidating how DNA damage is translated into a PafBC-dependent transcriptional response via sigma adaptation.

## MATERIALS AND METHODS

### Protein purification

All protein sequences of proteins purified and expressed in this study were derived from *M. smegmatis*.

Strep-tagged WT PafBC and all its variants were expressed with a C-terminal Strep-tag on PafC from an isopropyl-β-d-thiogalactopyranoside (IPTG)–inducible T7 promoter (pETDuet-1 vector). Plasmids encoding PafBC variants were generated from the WT by site-directed mutagenesis. The plasmids were transformed into *E. coli* Rosetta (DE3) cells (Invitrogen), and recombinant cells were grown at 37°C to an OD_600_ (optical density at 600 nm) of about 0.8 and then induced with 0.5 mM IPTG. Following further incubation for about 4 hours, cells were harvested by centrifugation (7000*g*, 15 min, 4°C). The pellet was frozen and stored at −20°C until further use. Cells were resuspended in a lysis buffer [50 mM Hepes-KOH (pH 7.8), 500 mM NaCl, and 1 mM DTT (dithiothreitol)] supplemented with 1 mM PMSF (phenylmethylsulfonyl fluoride) (Roche) and cOmplete protease inhibitor cocktail (Roche) and lysed using a microfluidizer [Microfluidics M-110L, chamber pressure of 11,000 psi (75,842,330 Pa), five passes]. The lysate was cleared by centrifugation (20,000 rpm = 47,810*g*, 30 min, 4°C), and the supernatant was supplied with DNase I (50 U/ml; Roche) and 1 mM MgCl_2_ and incubated at room temperature (RT) for 30 min. The sample was purified via a self-packed StrepTactin XT 4Flow high-capacity resin (IBA Lifesciences) column [buffer: 50 mM Hepes-KOH (pH 7.8), 500 mM NaCl, and 1 mM EDTA, supplemented with 50 mM biotin (IBA Lifesciences) for elution]. PafBC-containing fractions were pooled and concentrated using Amicon centrifugal filters (Merck Millipore). Last, the sample was subjected to gel filtration (Superdex 200 Increase, Cytiva) equilibrated in a storage buffer [25 mM Hepes-KOH (pH 7.8), 150 mM NaCl, 1 mM TCEP (tris(2-carboxyethyl)phosphine), and 5% v/v glycerol]. Fractions containing PafBC were pooled, concentrated using Amicon centrifugal filters (Merck Millipore), aliquoted, flash frozen in liquid nitrogen, and stored at −20°C until further use.

WT PafBC with an N-terminal TEV (tobacco etch virus) protease-cleavable His_6_-tag on PafB was expressed and purified as described before (*5*). Briefly, PafBC was expressed in *E. coli* Rosetta (DE3) cells, which were then harvested and lysed using a microfluidizer (Microfluidics M-110L). The cleared lysate was incubated with DNase I and subsequently purified via a self-packed Ni^2+^-affinity column (IMAC Sepharose 6 Fast Flow resin, Cytiva). PafBC-containing fractions were pooled, His_6_-tagged TEV protease was added, and the sample was dialyzed overnight. His_6_-tagged TEV protease was removed by Ni^2+^-affinity chromatography and PafBC collected in the flow-through. The sample was then further purified via ion-exchange chromatography (Source 30Q, Cytiva) followed by gel filtration (HiLoad Superdex 200 pg, Cytiva) in a storage buffer [10 mM Hepes-KOH (pH 7.8), 50 mM NaCl, and 1 mM TCEP]. Fractions containing PafBC were pooled, concentrated using Amicon centrifugal filters, aliquoted, flash frozen in liquid nitrogen, and stored at −20°C until further use.

RNAP was purified as previously described (*20*). Briefly, *M. smegmatis* SMR5 ∆*pafBC rpoC*-TwinStrep cells were grown, harvested, and lysed using a microfluidizer (Microfluidics M-110L). Removal of the insoluble material by centrifugation was followed by polyethyleneimine precipitation (0.35% v/v) to remove nucleic acid, and then total protein was precipitated with ammonium sulfate (70% w/v). After resolubilization, the sample was subjected to a self-packed StrepTactin XT 4Flow high-capacity resin column, and fractions containing RNAP were pooled and further purified via a HiPrep Heparin FF 16/10 column (Cytiva). Pooled fractions containing RNAP were applied to a gel filtration column (Superdex 200, Cytiva) run with a storage buffer [20 mM Hepes-KOH (pH 7.8), 100 mM potassium glutamate, 10 mM MgCl_2_, and 0.5 mM TCEP]. Fractions containing all core RNAP subunits were combined, concentrated using Amicon centrifugal filters, aliquoted, flash frozen in liquid nitrogen, and stored at −80°C until further use.

SigA, carrying an N-terminal His_6_-TEV-tag, and RbpA were coexpressed and copurified as described before (*20*). The final purified sample is therefore referred to as SigA-RbpA. In brief, SigA-RbpA was expressed in *E. coli* Rosetta (DE3) cells, which were then harvested and lysed using a microfluidizer (Microfluidics M-110L). The insoluble material was removed by centrifugation, and the supernatant was incubated with DNase I and subsequently purified by Ni^2+^-affinity chromatography. Pooled fractions containing SigA-RbpA were dialyzed in the presence of His_6_-tagged TEV protease, and cleaved Sig-RbpA was retrieved from the flow-through after reapplication to Ni^2+^-affinity chromatography. The sample was further purified by ion-exchange chromatography (Resource Q, Cytiva) followed by gel filtration (Superdex 200) in a storage buffer [20 mM Hepes-KOH (pH 7.8), 300 mM NaCl, 0.1 mM EDTA, 0.5 mM TCEP, and 5% v/v glycerol]. Fractions containing SigA-RbpA were pooled, concentrated using Amicon centrifugal filters, aliquoted, flash frozen in liquid nitrogen, and stored at −20°C until further use.

CarD was expressed as an N-terminal His_6_-thioredoxin-TEV fusion construct and purified as previously described (*20*). CarD was expressed in *E. coli* Rosetta (DE3) cells, which were then harvested and lysed using a microfluidizer (Microfluidics M-110L). The cleared lysate was incubated with DNase I and purified by Ni^2+^-affinity chromatography. Fractions containing CarD were combined and dialyzed in the presence of His_6_-tagged TEV protease. Cleaved CarD was obtained from the flow-through after reapplication to Ni^2+^-affinity chromatography, CarD-containing fractions were pooled and further purified via gel filtration (Superdex 75, Cytiva) in a storage buffer [20 mM Hepes-KOH (pH 7.8), 500 mM NaCl, 0.5 mM TCEP, and 5% v/v glycerol]. Fractions containing CarD were combined, concentrated using Amicon centrifugal filters, aliquoted, flash frozen in liquid nitrogen, and stored at −20°C until further use.

### Determination of protein concentrations and expected protein sizes

Protein concentrations were determined by absorbance at 280 nm. Molar extinction coefficients and expected protein sizes were calculated using the ExPASy ProtParam tool (*41*): ε_280 nm_ (WT PafBC, purification via His_6_-tag) = 78,840 M^−1^ cm^−1^, ε_280 nm_ (WT PafBC-Strep and PafBC-Strep variants containing arginine to alanine mutations) = 84,340 M^−1^ cm^−1^, ε_280 nm_ (PafBC-Strep B: Y196A) = 82,850 M^−1^ cm^−1^, ε_280 nm_ (PafBC-Strep C: W250A, Y254A, and Y255A) = 75,860 M^−1^ cm^−1^, ε_280 nm_ (RNAP: α_2_ββ′ω-σ^A^-RbpA) = 426,979 M^−1^ cm^−1^, ε_280 nm_ (SigA-RbpA) = 49,390 M^−1^ cm^−1^, and ε_280 nm_ (CarD) = 16,960 M^−1^ cm^−1^. Expected protein sizes: WT PafBC = 70 kDa, WT Strep-PafBC and Strep-PafBC variants = 71 kDa, RNAP(α_2_ββ′ω) = 362 kDa, SigA = 52 kDa, RbpA = 13 kDa, and CarD = 18 kDa.

### In vitro transcription assay

DNA templates containing the *M. smegmatis recA* promoter region followed by the sequence encoding dimeric Broccoli and a tRNA scaffold according to Filonov *et al.* (*22*) were cloned into a pFLAG_attp vector (fig. S1A). For the PafBC-dependent promoter template (table S2), the LexA/RecA-dependent promoter was replaced by a stretch of coding sequence of the mouse histone H2AB2 of identical size and similar GC content, as previously described in (*5*). For the promoterless template (table S2), the entire promoter region containing both the PafBC-dependent and the LexA/RecA-dependent promoter was replaced by a stretch of coding sequence of the *M. smegmatis* mycobacterial proteasome adenosine triphosphatase (Mpa) of identical size and similar GC content. In all transcription assays with PafBC-dependent promoter templates, except those shown in Fig. 1C, the promoter template included the first 36 bases of the *recA* gene upstream of the RNA aptamer sequence [see table S2: PafBC-dependent promoter template (long)]. For the transcription assays shown in Fig. 1C, a shorter DNA template was used, in which the distance between the PafBC-dependent promoter and the start of the RNA aptamer sequence had been minimized [see table S2: PafBC-dependent promoter template (short)], likely resulting in more efficient folding of the RNA aptamer and thus slightly higher fluorescence levels. The DNA templates were polymerase chain reaction (PCR) amplified using Q5 High-Fidelity DNA Polymerase (NEB), digested with DpnI (NEB) and ExoI (NEB) for 1 hour at 37°C, and subsequently purified using RNAClean XP magnetic beads (Beckman Coulter). After length verification by agarose gel electrophoresis, DNA concentration was determined by absorbance at 260 nm, and the DNA templates were frozen and stored at −20°C until further use. Lyophilized ssDNA and ssRNA oligonucleotides (Microsynth) were dissolved in water, the concentration was determined by absorbance at 260 nm, and the ssDNA was frozen and stored at −20°C until further use. Nucleotide second messengers including cAMP, cGMP (cyclic GMP), cyclic-di-AMP (cyclic diadenosine monophosphate), cyclic-di-GMP (cyclic diguanosine monophosphate), 2′,3′-cGAMP and 3′,3′-cGAMP, ppGpp (guanosine-3′,5′-bisdiphosphate), and pppGpp (all derived from Jena Bioscience) were dissolved in water and then frozen and stored at −20°C until further use.

The in vitro transcription reactions were prepared as 20-μl reactions, which included 20 μM DFHBI-1T (Jena Bioscience), 1 mM rNTPs (Ribonucleotide Solution Mix, NEB), 0.05 μM DNA template, 0.5 μM RNAP, 2.5 μM SigA-RbpA, 10 μM CarD, 5 μM PafBC, and 20 μM ssDNA/ssRNA/nucleotide second messengers in a reaction buffer [20 mM Hepes-KOH (pH 7.8), 12 mM MgCl_2_, 50 mM KCl, and 1 mM DTT] supplemented with RiboLock RNase Inhibitor (0.5 U/μl; Thermo Fisher Scientific) and bovine serum album (BSA) (0.05 mg/ml; Thermo Fisher Scientific). Unless otherwise specified in the figure legend, the PafBC-dependent promoter template was used for transcription reactions. Strep-tagged PafBC was used for the transcription assay comparing WT PafBC and the WYL arginine PafBC variant shown in Fig. 1E, whereas tag-free PafBC (see purification with N-terminal TEV-cleavable His_6_-tag on PafB) was used for all other transcription assays. A mastermix containing the components identical in all reactions (except RNAP) was prepared first, and the remaining components that differ between reactions, as well as RNAP, were provided in 0.2-ml tubes. The mastermix was added to each reaction, and the reactions were mixed thoroughly, transferred to black 96-well PCR plates (Bio-Rad), and covered with a transparent seal (Bio-Rad). Control reactions were prepared similarly but included only the mastermix. The plate was centrifuged (2500 rpm = 1258*g*, 1 min, RT), and fluorescence (excitation: 450 to 490 nm; emission: 510 to 530 nm) was measured every 30 s at 37°C for 1 hour using a quantitative PCR (qPCR) instrument (CFX96 Touch Real-Time PCR Detection System, Bio-Rad). For evaluation, the fluorescence of the control reaction was subtracted from the fluorescence of the tested reactions. For the comparison of PafBC-dependent transcription upon addition of ExoI or ExoI storage buffer [10 mM tris-HCl (pH 7.5), 100 mM NaCl, 5 mM β-mercaptoethanol, 0.5 mM EDTA, BSA (0.1 mg/ml), and 50% v/v glycerol], the plate was taken from the qPCR instrument after 15 min, the seal was removed, and 2.5 μl of ExoI (20,000 U/ml = 50 U) or ExoI storage buffer was added to the reactions. Afterward, the plate was resealed and the fluorescence measurement continued. Each reaction was performed in at least three independent replicates, and data are represented as mean with SD.

### EMSA and native PAGE analysis

Lyophilized unlabeled and 6-carboxyfluorescein (FAM)–labeled oligonucleotides (Microsynth) were dissolved in water or 10 mM Hepes-KOH (pH 7.8), respectively. The DNA concentration was determined by absorbance at 260 nm, and the oligonucleotides were frozen and stored at −20°C until further use. The blocked end ssDNA (see table S2) was designed to comprise a dT12 ssDNA stretch flanked by two identical DNA hairpin loops [each with a 14–base pair (bp) stem and a 5-base loop]. The corresponding control hairpin DNA comprises just a single DNA hairpin (14-bp stem and a 5-base loop). The secondary structure of both constructs was predicted using the VectorBuilder tool (https://en.vectorbuilder.com/tool/dna-secondary-structure.html). The blocked end ssDNA and the hairpin DNA were freshly annealed by heating at 95°C for 10 min, followed by slow temperature decrease to RT (1°C/min) in an annealing buffer [10 mM Hepes-KOH (pH 7.5), 50 mM NaCl, and 1 mM EDTA].

EMSAs studying the binding of PafBC to ssDNA were performed as follows. Strep-tagged PafBC or PafBC variants (0 to 16 μM) were incubated with 10 nM fluorescently labeled dT20 ssDNA or dT12 blocked end ssDNA in a reaction buffer [22.5 mM Hepes-KOH (pH 7.8), 135 mM NaCl, 20 mM KCl, 5 mM MgCl_2_, 1 mM DTT, 0.7 mM TCEP, 2.5% w/v Ficoll 400K, and 4.5% v/v glycerol] at 37°C for 15 min. Samples were loaded onto native gels [8% acrylamide/bis-acrylamide (19:1) and 30% v/v triethylene glycol in 0.5x TB buffer: 45 mM Tris base and 45 mM boric acid] and run in 0.5x TB buffer for 70 to 90 min at 120 V at RT. Afterward, the gel was imaged using a Sapphire Biomolecular Scanner (Azure Biosystems) for visualization of the FAM-labeled DNA. Each experiment was performed in at least three independent replicates.

EMSAs studying the complex formation of RNAP-σ^A^ and PafBC were performed as follows. Fluorescently labeled promoter dsDNA scaffolds were freshly prepared by annealing an FAM-labeled oligonucleotide with the complementary oligonucleotide in an annealing buffer [10 mM Hepes-KOH (pH 7.5), 50 mM NaCl, and 1 mM EDTA] by heating at 95°C for 10 min, followed by slow temperature decrease to RT (1°C/min). The PafBC-specific promoter scaffold is based on the promoter of the *M. smegmatis recA* gene, the housekeeping promoter scaffold is based on the *M. smegmatis* AP3 promoter, and the nonspecific DNA scaffold is based on a stretch of coding sequence of the *M. smegmatis gyrB* gene (see table S2). RNAP (0.5 μM), SigA-RbpA (1.5 μM), CarD (1.5 μM), Strep-tagged PafBC or PafBC variants (3 μM), FAM-labeled promoter dsDNA (0.2 μM), and ssDNA (5 μM) were mixed in a reaction buffer [20 mM Hepes-NaOH (pH 8), 6 mM Hepes-KOH (pH 7.8), 36 mM NaCl, 100 mM potassium acetate, 10 mM MgCl_2_, 2 mM DTT, 0.24 mM TCEP, and 1.2% v/v glycerol] when indicated. For the EMSA shown in fig. S4D, the ssDNA used for cryo-EM sample preparation was added (5′-TTGTTGTTGTT-3′); for all other EMSAs studying the RNAP-PafBC complex formation, dT12 ssDNA was used. Samples were incubated at 37°C for 30 min and then loaded onto native gels [4.5% acrylamide/bis-acrylamide (37.5:1) and 4% v/v glycerol in 1x TBE buffer: 89 mM Tris base, 89 mM boric acid, and 2 mM EDTA] and run in 1x TBE buffer for 60 min at 15 mA per gel at 4°C. Afterward, the gel was imaged using a Sapphire Biomolecular Scanner (Azure Biosystems) for visualization of the FAM-labeled DNA or Coomassie stained for protein visualization. Each experiment was performed in at least three independent replicates.

### Analytical gel filtration experiments for determining the ssDNA-to-PafBC stoichiometry

Samples containing PafBC at a constant concentration of 20 μM and increasing amounts of dT20 ssDNA (0, 4, 8, 12, 16, 20, 24, 28, 32, 36, and 40 μM) were prepared in a reaction buffer [20 mM Hepes-KOH (pH 7.8), 15 mM NaCl, 2 mM MgCl_2_, and 1 mM DTT] and incubated at 37°C for 15 min. Samples were loaded onto a Superdex 200 Increase 5/150 GL column (Cytiva) connected to a high-performance liquid chromatography system (Agilent Technologies, 11 Series) running with a flow rate of 0.45 ml/min. Chromatograms were recorded at 280 nm. The peak areas corresponding to the sum of PafBC and the ssDNA:PafBC complex were determined using OriginPro 2023. The peak area increase over the PafBC peak in the absence of ssDNA represents the amount of ssDNA in the complex because the PafBC concentration remains constant. The increase was plotted against the respective molar ratios of ssDNA:PafBC. The first and last five measurements corresponding to the data points before and after saturation, respectively, were linearly fitted, and the intercept between the linear regression curves yielded the ssDNA-to-PafBC stoichiometry.

### Multiple sequence alignment

PafB and PafC amino acid sequences from several actinobacterial species as well as the DriD sequence from *C. crescentus* were compiled, aligned in ClustalW (*42*, *43*), and visualized with Jalview (*44*).

### Cryo-EM sample preparation and data collection

Lyophilized oligonucleotides (Microsynth) were dissolved in water, the concentration was determined by absorbance at 260 nm, and the oligonucleotides were frozen and stored at −20°C until further use. PafBC-dependent promoter DNA [as described in (*20*)] was freshly prepared by annealing complementary oligonucleotides in an annealing buffer [10 mM Hepes-KOH (pH 7.5), 50 mM NaCl, and 1 mM EDTA] by incubation at 95°C for 10 min, followed by slow temperature decrease to RT (1°C/min). RNAP (1 μM), SigA-RbpA (3 μM), PafBC (4 μM), ssDNA (32 μM; 5′-TTGTTGTTGTT-3′), and promoter dsDNA (1.5 μM) were mixed in a reaction buffer [20 mM Hepes-NaOH (pH 8), 100 mM potassium acetate, 10 mM MgCl_2_, and 0.5 mM TCEP) and incubated for 30 min at 37°C. The sample was cross-linked by incubation with 0.5% formaldehyde (Fisher Scientific) for 1 hour, and the residual cross-linker was subsequently quenched by the addition of tris-HCl (pH 8) to an ~2.4 molar excess over the cross-linker. Afterward, the sample was purified by gel filtration (Superose 6 Increase, Cytiva) equilibrated in a reaction buffer. Fractions containing RNAP-σ^A^ and the RNAP-PafBC complex were pooled and concentrated using Amicon centrifugal filters to ~0.5 mg/ml.

The cross-linked sample (4 μl) was applied to Quantifoil Cu 300 R2/2 holey carbon grids that were coated with an ~1-nm continuous carbon film and glow discharged (15 s at 15 mA) prior to use. After incubation for 30 s, the grids were blotted for 1 s, and the sample was vitrified in a liquid ethane/propane mixture using a Vitrobot Mark IV (Thermo Fisher Scientific) at 4°C and 95% humidity. Data were collected on a Titan Krios transmission electron microscope (Thermo Fisher Scientific) operated at 300 kV using the Gatan K3 direct electron detector in counting super-resolution mode and a GIF-Quantum energy filter (Gatan) with a slit width of 20 eV. Images were recorded with a total electron dose of 81 and 76 e^−^/Å^2^, respectively, applied over 40 frames, a targeted defocus set between −1.0 and −3.0 μm, and ×105,000 magnification resulting in a pixel size of 0.84 Å/px.

Initially, a cryo-EM sample including CarD was prepared as described above but with CarD added to a final concentration of 3 μM. From this sample, one dataset was collected, and a brief overview of the processing is provided in the legend to fig. S4. As the presence of CarD increased the particle heterogeneity, it was not included in the second cryo-EM sample. The CarD dataset was not used further; therefore, the following sections focus only on the second cryo-EM sample containing RNAP, SigA-RbpA, PafBC, promoter DNA, and ssDNA.

### Cryo-EM data processing

Two datasets were collected for the RNAP-PafBC transcription initiation complex and initially processed separately following an analogous workflow. All processing steps were performed in cryoSPARC (*45*). Following motion correction, contrast transfer function estimation, and removal of poor-quality micrographs, 30,897 and 36,856 micrographs of datasets 1 and 2, respectively, were selected. Particles were picked from the selected micrographs using Topaz (*46*). A total of 31,946,696 particles (14,019,807 and 17,926,889 from datasets 1 and 2, respectively) were extracted with a 100-px box size (4.03 Å/px) and subjected to multiple rounds of 2D classification to remove false-positive picks. Particles from selected 2D class averages (4,785,557 and 7,785,719 from datasets 1 and 2, respectively) were then subjected to 3D heterogeneous refinement without a solvent mask. As initial reference, a volume low-pass filtered to 20 Å with density visible for both RNAP and PafBC obtained from our first cryo-EM sample including CarD was provided. Classes containing the best-resolved density for PafBC were selected (2′024’283 and 3′072’522 from datasets 1 and 2, respectively) before two more rounds of classification by 3D heterogeneous refinement (same parameters) were performed. Subsequently, the classes with good densities for both PafBC and RNAP from both datasets were combined (1,196,412 particles), particles were re-extracted with a 256-px box size (1.58 Å/px) and subjected to another 3D heterogeneous refinement without a solvent mask. Selected particles (897,174) were then globally aligned on RNAP by nonuniform refinement. Subsequent no-alignment 3D classification (10 classes, mask on RNAP, 470,770 particles selected) followed by nonuniform refinement yielded map_RNAP_ at 3.3-Å global resolution. PafBC processing was continued by 3D classification (without alignment) focused on PafBC (target resolution: 6 Å, 15 classes). Particles from classes with promising PafBC density (514,889 particles) were pooled and once again globally aligned on RNAP by nonuniform refinement. Afterward, subtraction of the RNAP signal was performed and the resulting signal-subtracted particles were locally 3D refined. Particles were then downsampled to 2.9 Å/px and subjected to 3D variability analysis (mask on PafBC only) requesting three modes and six clusters. This was followed by another 3D variability analysis (mask on PafBC and bound dsDNA) requesting five modes and six clusters, ultimately yielding 154,302 particles that underwent local 3D refinement. Using original unsubtracted particles with alignments determined in the last local 3D refinement, another local 3D refinement on PafBC was performed. Last, particles downsampled to 3.1 Å/px were used in the 3D flexible refinement (latent dimensions: 4; latent centering strength: 1) (*47*), resolving map_PafBC_ at 4-Å global resolution. In addition, OccuPy (*48*) was used for amplification of the PafBC density and solvent suppression. Figure 3A shows map_PafBC_ generated by OccuPy.

### Model building

The AlphaFold (*49*) model of PafBC from *M. smegmatis* was split into multiple domains (HTH/linker-BC, WYL-B, WYL-C, and WCX-BC), which were then rigid-body fitted into map_PafBC_ using UCSF ChimeraX (*50*). The individual models were subjected to five cycles of real-space refinement in PHENIX (*51*) with standard parameters, using protein secondary structure and side-chain rotamer restraints. Subsequently, the individual domain models were reassembled into the full PafBC structure in COOT (*52*), which was then real-space refined in PHENIX with the same parameters as before. Following manual real-space refinement in COOT to reduce the clashscore and rotamer outliers, the model was subjected to another five cycles of real-space refinement in PHENIX using the same parameters. PHENIX validation statistics of the final PafBC model are shown in table S1. The Fo-Fc difference density map was generated with the phenix.real_space_diff_map command in Phenix at 4-Å resolution using the map_PafBC_ (amplified in OccuPy) and the refined PafBC model.

The RNAP complex model was built on the basis of the available RNAP-PafBC structure containing the HTH domains of PafBC [Protein Data Bank (PDB): 7P5X; (*20*)]. First, the RNAP holoenzyme (without PafBC) was real-space refined against map_RNAP_ in PHENIX with standard parameters, using protein secondary structure and side chain rotamer restraints. Then, map_PafBC_ was fitted into the HTH domain densities resolved in the map_RNAP_. The two maps were subsequently combined in UCSF ChimeraX (volume add) to generate the composite map of the full RNAP-PafBC complex. Last, after the refined PafBC model was rigid-body fitted into the composite map using UCSF ChimeraX, the resulting RNAP-PafBC complex underwent five cycles of real-space refinement against the composite map in PHENIX as two rigid bodies (RNAP holoenzyme and PafBC).

All models were validated using the comprehensive validation tool in PHENIX with MolProbity (*53*) providing model statistics. All cryo-EM maps and models were visualized using UCSF ChimeraX.
