# Supplementary material for: Single-stranded DNA binding to the transcription factor PafBC triggers the mycobacterial DNA damage response
Source: Sci Adv. 2025 Feb 7;11(6):eadq9054. doi: 10.1126/sciadv.adq9054 (PMC11804915; doi:10.1126/sciadv.adq9054)
Supplement: Supplementary file 1 — Figs. S1 to S9 Tables S1 and S2 [file sciadv.adq9054_sm.pdf]

Science Advances
AAAS

Supplementary Materials for

Single-stranded DNA binding to the transcription factor PafBC triggers the mycobacterial DNA damage response

Charlotte M. Schilling *et al.*

Corresponding author: Eilika Weber-Ban, eilika@mol.biol.ethz.ch

Sci. Adv. **11**, eadq9054 (2025)
DOI: 10.1126/sciadv.adq9054

This PDF file includes:

Figs. S1 to S9
Tables S1 and S2

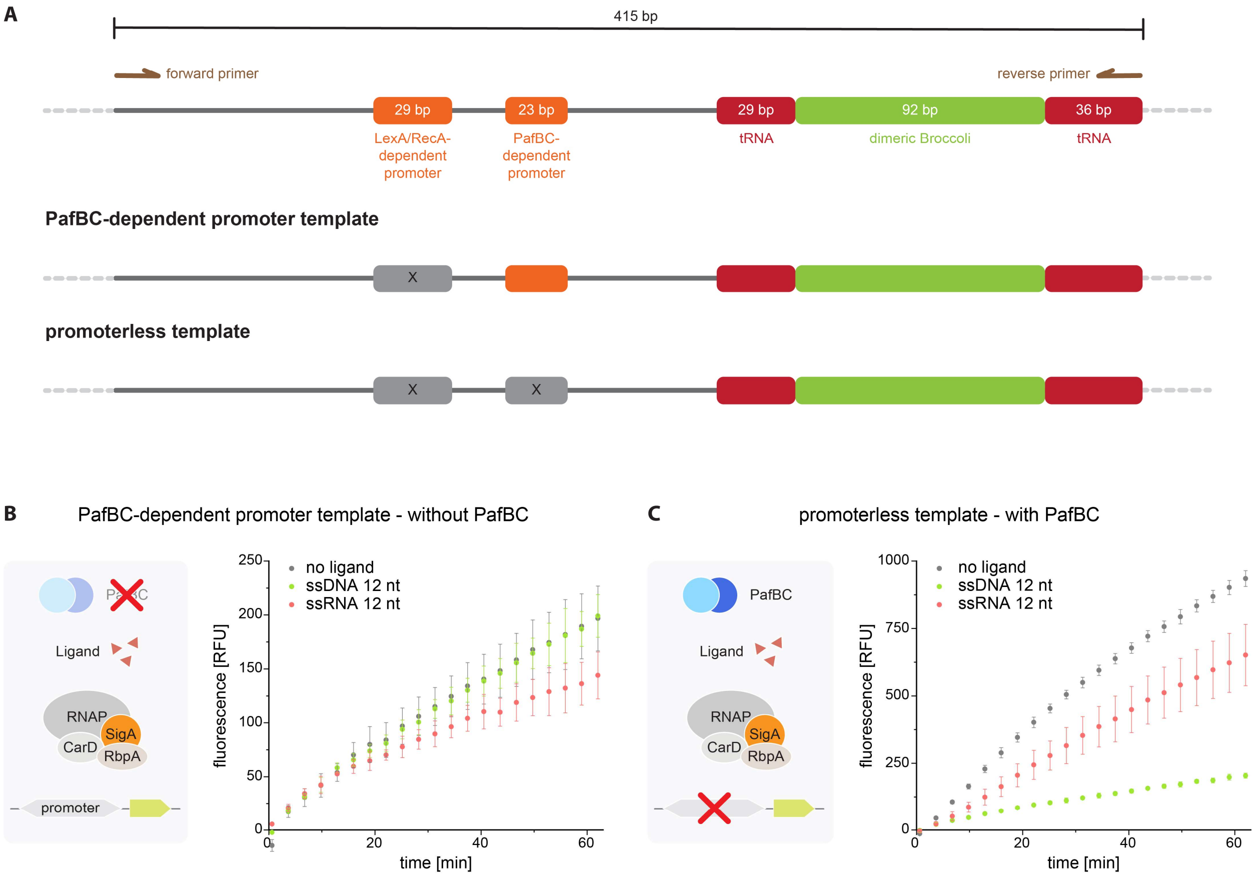

Fig. S1. The effect of ssDNA on transcription is absent in transcription reactions lacking PafBC or when DNA templates lacking the PafBC-dependent promoter are used. (**A**) Overview of the dsDNA templates for *in vitro* transcription showing the LexA/RecA-dependent and the PafBC-dependent promoter in orange, the RNA aptamer sequence (dimeric Broccoli) in green, and the tRNA scaffold for increased folding efficiency of the RNA aptamer in red. (**B**) *In vitro* transcription assay comparing the effect of the addition of ssDNA (green: 5'-GTACAGTCGTAC-3') or ssRNA (red: 5'-GUACAGUCGUAC-3') in the absence of PafBC. The RNA aptamer sequence was under the control of the PafBC-dependent promoter. (**C**) Comparison of transcription levels measured upon addition of ssDNA (green: 5'-GTACAGTCGTAC-3') or ssRNA (red: 5'-GUACAGUCGUAC-3') to a reaction containing the promoterless dsDNA template encoding the RNA aptamer. Addition of ssDNA results in decreased basal transcription, likely due to the high excess of ssDNA over promoter dsDNA and nonspecific binding of RNAP-σ^A to DNA.

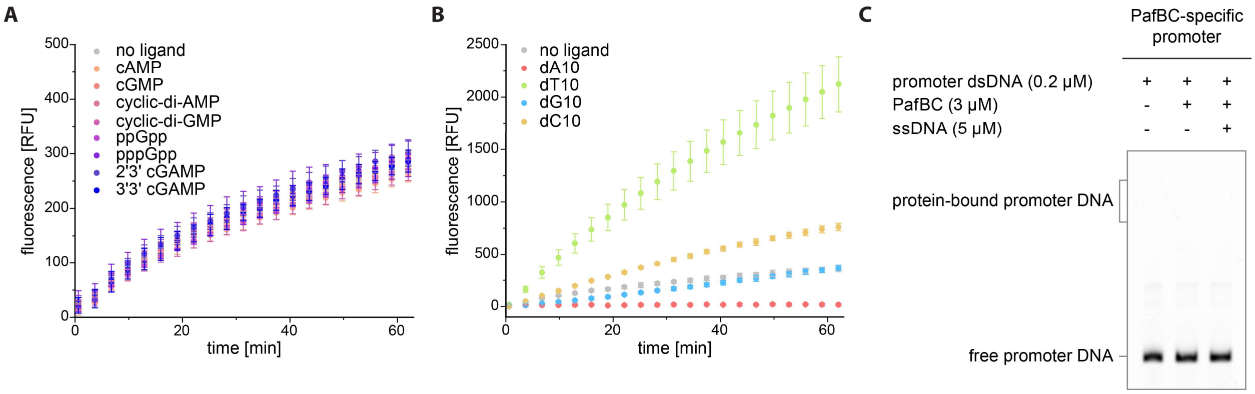

Fig. S2. PafBC is activated most efficiently by poly-thymidine ssDNA and not activated by nucleotide second messengers. (**A**) The activation of PafBC by various nucleotide second messengers (20 μM) was evaluated using the *in vitro* transcription assay. (**B**) Comparison of PafBC activation in the presence of 10 nt long ssDNAs containing only either adenine, thymine, guanine, or cytosine (dA, dT, dG, dC, respectively). The addition of dA10 ssDNA results in abolished transcription, likely due to nonspecific binding of RNAP-σ^A to the ssDNA. (**C**) Binding of PafBC to PafBC-specific promoter dsDNA without a preformed transcription bubble at the -10 region was examined using EMSAs. PafBC was added to the fluorescently labeled promoter DNA in the presence or absence of dT12 ssDNA.

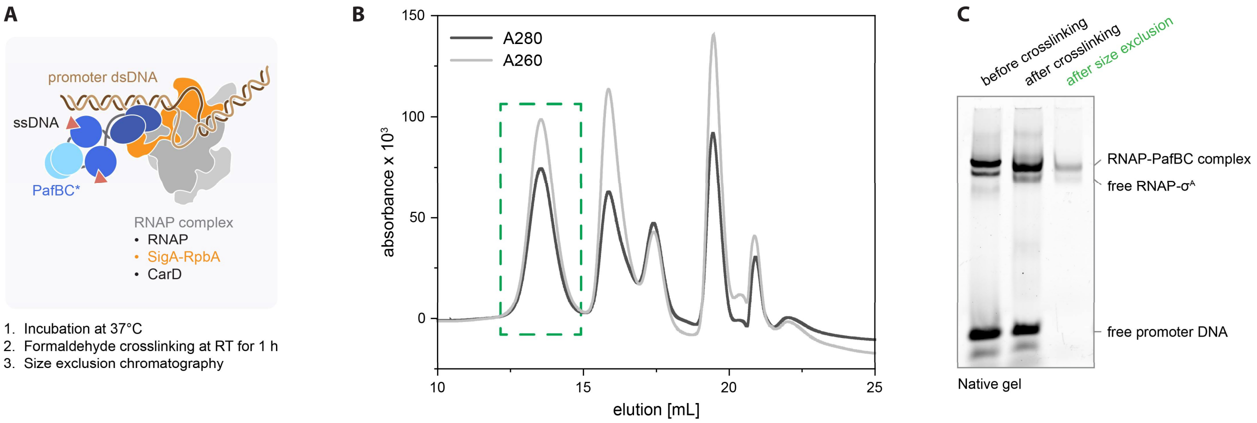

Fig. S3. Cryo-EM sample preparation. (**A**) Overview of the cryo-EM sample preparation. The transcription initiation complex was initially assembled in the presence of CarD. However, we could show that CarD is not required for stabilization of the open complex in our sample, due to the preformed transcription bubble within the promoter DNA scaffold. Therefore, CarD was excluded from the final cryo-EM sample that was used to solve the PafBC structure. (**B**) Representative size exclusion chromatography (SEC) run of the crosslinked RNAP-PafBC complex. (**C**) Crosslinking efficiency with formaldehyde of the RNAP-PafBC complex was initially analyzed by EMSA with fluorescently labeled promoter DNA. The label "free RNAP-σ^A" corresponds to RNAP-σ^A that is bound to promoter DNA but is not associated with PafBC. The third gel lane corresponds to the crosslinked complex isolated via SEC (green box in B).

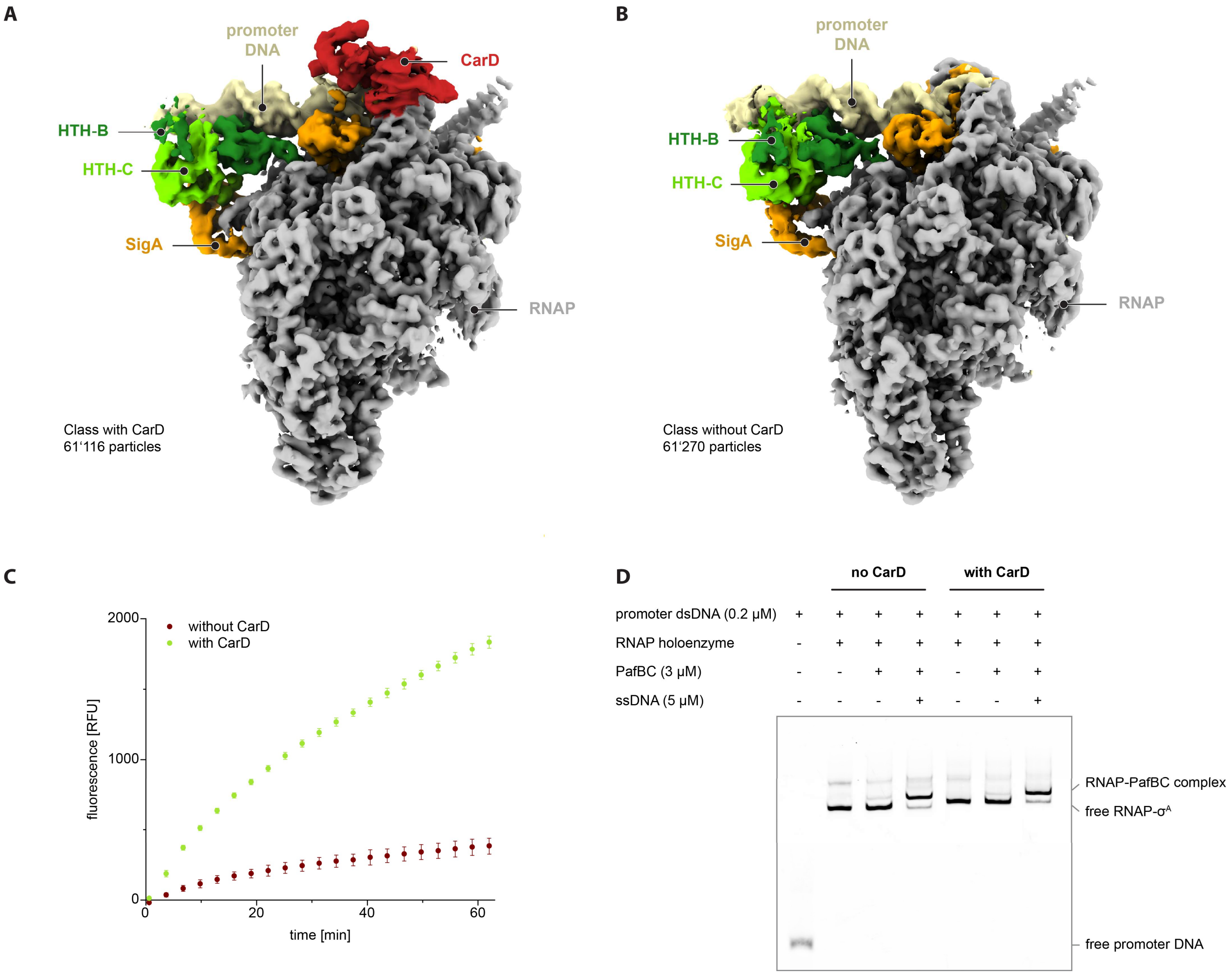

Fig. S4. CarD stimulates PafBC-dependent transcription. (**A**, **B**) Representative cryo-EM maps obtained for the crosslinked RNAP-PafBC sample containing CarD. For this sample, one dataset was collected and processed in cryoSPARC. Following motion correction, CTF estimation and exposure curation, particles were picked using Topaz, extracted, and subjected to multiple rounds of 2D classification. Initial 3D variability analysis revealed two populations of particles, one with and one without CarD. Therefore, 3D heterogeneous refinement was performed on the particles corresponding to the selected 2D class averages, with input volumes derived from 3D variability analysis with and without CarD. Subsequently, no-alignment 3D classification focused on CarD was used to split the particles into two populations, comprising either RNAP without or with CarD bound. Following non-uniform refinement focused on RNAP, both particle classes were individually subjected to no-alignment 3D classification focused on PafBC to determine whether the presence of CarD affected the binding of PafBC. Both populations (± CarD) contained similar fractions of PafBC bound to RNAP-σ^A (~65% of particles contained PafBC in both populations). Representative 3D class averages of both populations are depicted here, having both PafBC (green, HTH domains resolved) and CarD (red) bound shown on the left, or only PafBC bound

shown on the right. (**C**) PafBC-dependent transcription upon addition of ssDNA (5’-GTACAGTCGTAC-3’) was measured in the presence (green) or absence (brown) of CarD (10 µM). (**D**) RNAP-PafBC complex formation with and without CarD was evaluated by EMSAs using fluorescently labeled PafBC-specific promoter DNA which contained a preformed transcription bubble at the -10 region of the promoter. RNAP holoenzyme corresponds to 0.5 µM RNAP supplemented with 1.5 µM SigA-RbpA. CarD was added to 1.5 µM when indicated. Reactions were compared in the absence and presence of dT12 ssDNA. The label “free RNAP-σ^A” corresponds to RNAP-σ^A that is bound to promoter DNA but is not associated with PafBC.

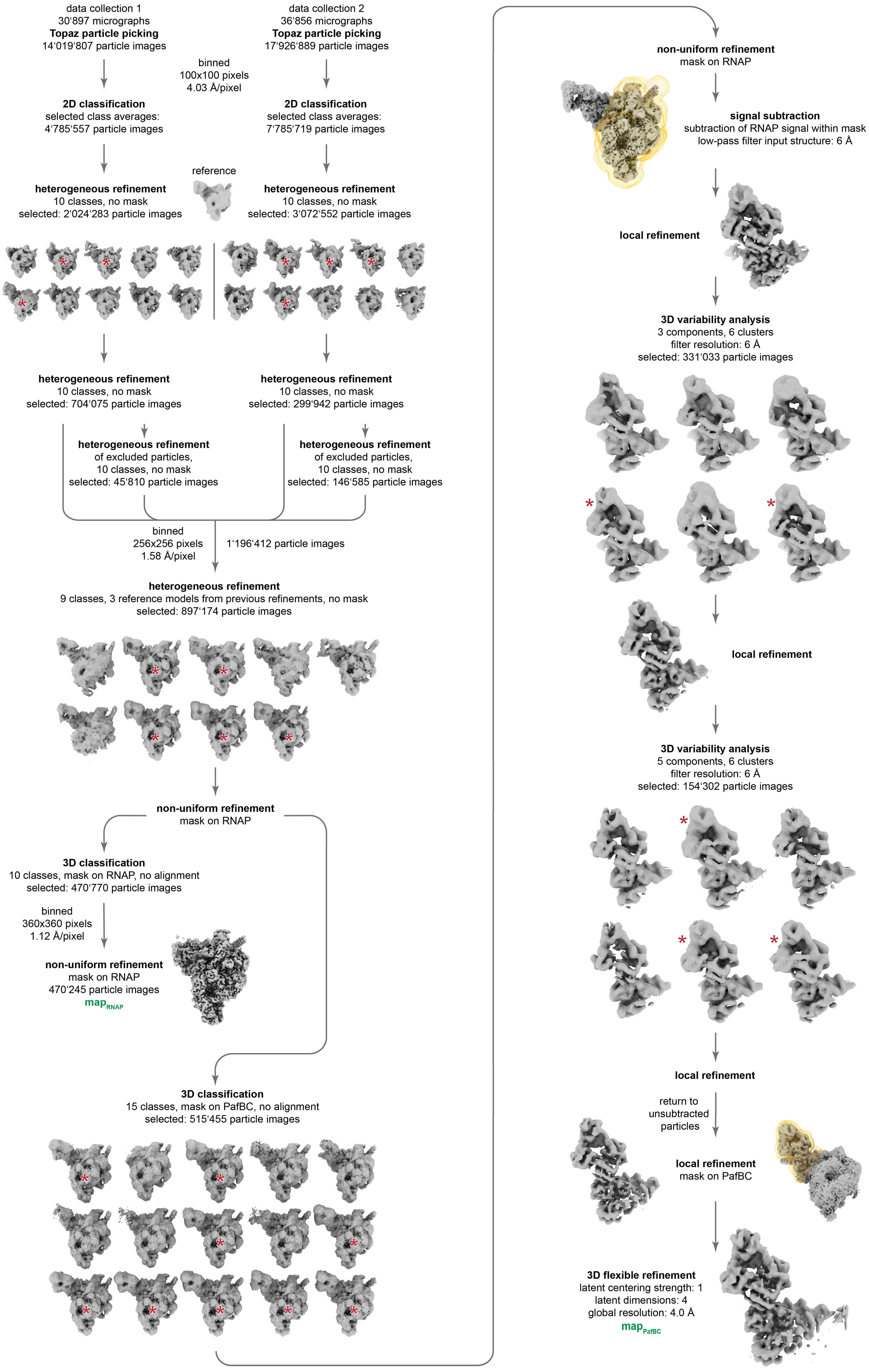

data collection 1
30‘897 micrographs
Topaz particle picking
14‘019‘807 particle images
data collection 2
36‘856 micrographs
Topaz particle picking
17‘926‘889 particle images
binned
100x100 pixels
4.03 Å/pixel
2D classification
selected class averages:
4‘785‘557 particle images
2D classification
selected class averages:
7‘785‘719 particle images
reference
heterogeneous refinement
10 classes, no mask
selected: 2‘024‘283 particle images
heterogeneous refinement
10 classes, no mask
selected: 3‘072‘552 particle images
*
heterogeneous refinement
10 classes, no mask
selected: 704‘075 particle images
heterogeneous refinement
10 classes, no mask
selected: 299‘942 particle images
heterogeneous refinement
of excluded particles,
10 classes, no mask
selected: 45‘810 particle images
heterogeneous refinement
of excluded particles,
10 classes, no mask
selected: 146‘585 particle images
binned
256x256 pixels
1.58 Å/pixel
1‘196‘412 particle images
heterogeneous refinement
9 classes, 3 reference models from previous refinements, no mask
selected: 897‘174 particle images
non-uniform refinement
mask on RNAP
3D classification
10 classes, mask on RNAP, no alignment
selected: 470‘770 particle images
binned
360x360 pixels
1.12 Å/pixel
non-uniform refinement
mask on RNAP
470‘245 particle images
map RNAP
3D classification
15 classes, mask on PafBC, no alignment
selected: 515‘455 particle images
non-uniform refinement
mask on RNAP
signal subtraction
subtraction of RNAP signal within mask
low-pass filter input structure: 6 Å
local refinement
3D variability analysis
3 components, 6 clusters
filter resolution: 6 Å
selected: 331‘033 particle images
local refinement
3D variability analysis
5 components, 6 clusters
filter resolution: 6 Å
selected: 154‘302 particle images
local refinement
return to
unsubtracted
particles
local refinement
mask on PafBC
3D flexible refinement
latent centering strength: 1
latent dimensions: 4
global resolution: 4.0 Å
map PafBC

Fig. S5. Cryo-EM processing scheme. Two datasets were collected and processed in cryoSPARC. Early processing was performed separately for the two datasets. Following motion correction, CTF estimation and selection of good micrographs, particles were picked using Topaz. Extracted particles were subjected to multiple rounds of 2D classification before 3D heterogeneous refinement on the particles corresponding to the selected 2D class averages was performed. Classes containing the strongest density for PafBC were selected (marked with red asterisks) and subjected to two more rounds of classification by 3D heterogeneous refinement. Subsequently, the best classes (with good densities for both PafBC and RNAP) from both datasets were combined and subjected to another 3D heterogenous refinement. Following non-uniform refinement focused on RNAP, no alignment 3D classification on PafBC was carried out. Classes with promising PafBC density were pooled and subjected to another non-uniform refinement focused on RNAP before subtracting the RNAP signal (orange mask). After local 3D refinement of the signal-subtracted particles, two rounds of 3D variability analysis followed by local refinement of the selected clusters were carried out. Finally, local refinement on PafBC using original particles (containing the RNAP signal; using alignments of the signal-subtracted particles) and subsequent 3D flexible refinement were conducted, yielding the final PafBC map (map_{PafBC}) at 4.0 Å global resolution.

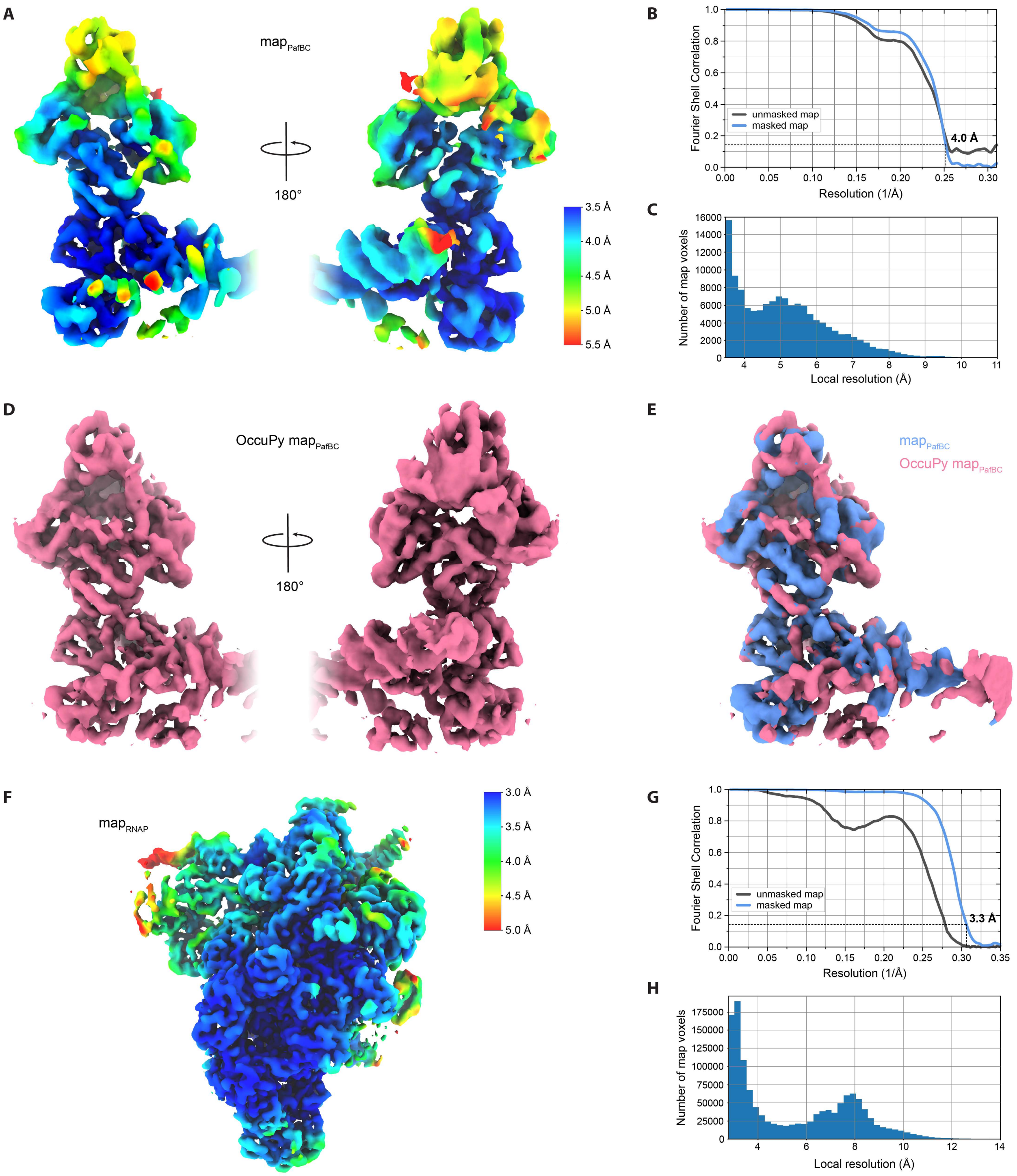
A
map$_{PafBC}$
180°
3.5 Å
4.0 Å
4.5 Å
5.0 Å
5.5 Å
B
Fourier Shell Correlation
unmasked map
masked map
4.0 Å
Resolution (1/Å)
C
Number of map voxels
Local resolution (Å)
D
OccuPy map$_{PafBC}$
180°
E
map$_{PafBC}$
OccuPy map$_{PafBC}$
F
map$_{RNAP}$
3.0 Å
3.5 Å
4.0 Å
4.5 Å
5.0 Å
G
Fourier Shell Correlation
unmasked map
masked map
3.3 Å
Resolution (1/Å)
H
Number of map voxels
Local resolution (Å)

Fig. S6. Supplementary data on the cryo-EM single-particle analysis of the RNAP-PafBC complex. (**A**) Unsharpened map_{PafBC} colored according to the local resolution estimated in cryoSPARC. The color key is shown on the bottom right. (**B**) Fourier shell correlation (FSC) plot of map_{PafBC}. The dotted line marks the FSC = 0.143 cutoff. (**C**) Local resolution histogram of map_{PafBC} (FSC = 0.500). (**D**) Map_{PafBC} after signal amplification and solvent suppression using OccuPy. (**E**) Superimposed PafBC maps before (blue) and after (pink) density amplification in OccuPy. (**F**) Unsharpened map_{RNAP} colored according to the local resolution estimated in cryoSPARC. The color key is shown on the top right. (**G**) FSC plot of map_{RNAP}. The dotted line marks the FSC = 0.143 cutoff. (**H**) Local resolution histogram of map_{RNAP} (FSC = 0.500).

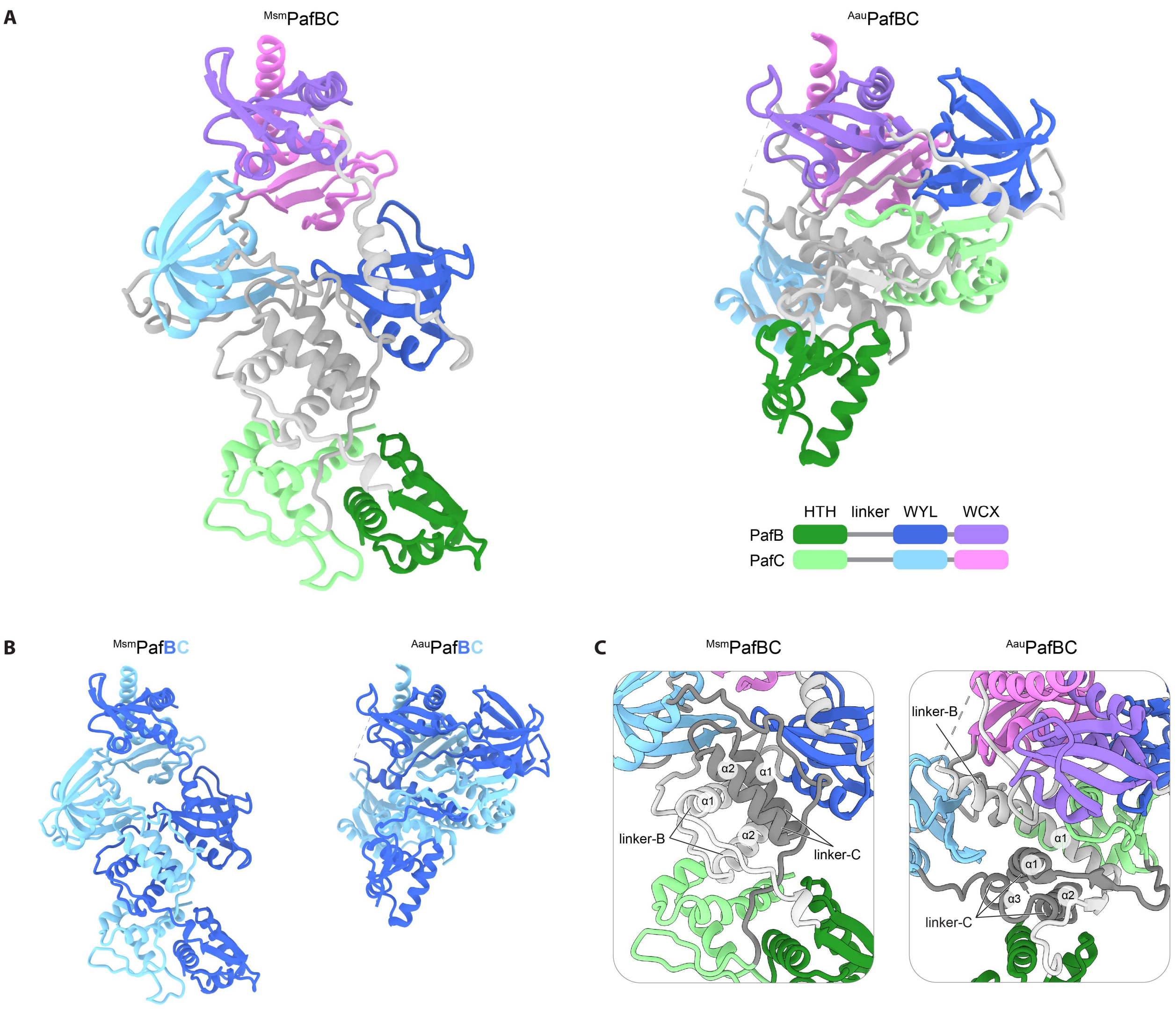

Fig. S7. Comparison of the *M. smegmatis* PafBC cryo-EM structure and the *A. aurescens* PafBC crystal structure (PDB: 6SJ9). (**A**) ^{Msm}PafBC and ^{Aau}PafBC structures colored according to the domain organization depicted on the bottom right. ^{Aau}PafBC is a naturally occurring fusion of the heterodimer into one polypeptide chain. (**B**) Structures of ^{Msm}PafBC and ^{Aau}PafBC with PafB and PafC colored in dark and light blue, respectively. (**C**) Close-up view of the differently structured linkers connecting the HTH and WYL domains in ^{Msm}PafBC and ^{Aau}PafBC. The PafB and PafC linkers are colored in light and dark grey, respectively.

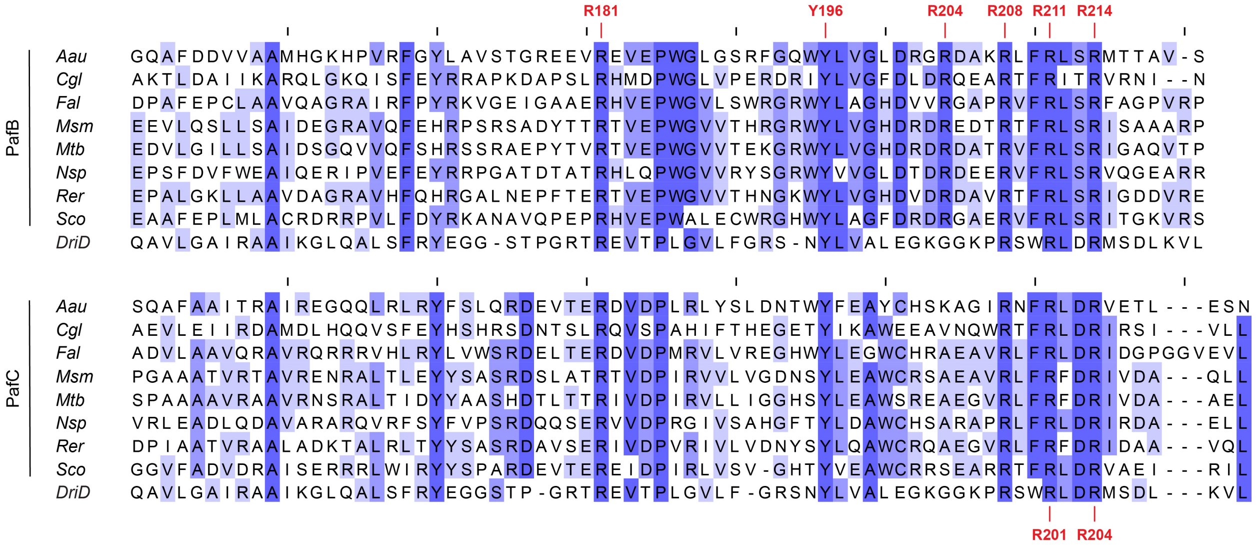

Fig. S8. Sequence alignment of PafB and PafC WYL domains from different orthologs and the WYL domain of *C. crescentus* DriD. *Aau* = *Arthrobacter aurescens* (A0A4Y3NDN0), *Cgl* = *Corynebacterium glutamicum* (Q8NQE2, Q8NQE3), *Fal* = *Frankia alni* (Q0RLT0, Q0RLS9), *Msm* = *Mycobacterium smegmatis* (I7G3U5, A0QZ41), *Mtb* = *Mycobacterium tuberculosis* (P9WIM1, P9WIL9), *Nsp* = *Nocardioides sp.* (A1SK18, A1SK19), *Rer* = *Rhodococcus erythropolis* (C0ZZU3, C0ZZU2), *Sco* = *Streptomyces coelicolor* (Q9RJ64, Q9RJ65), DriD from *Ccr* = *Caulobacter crescentus* (Q9A999). UniProt accession numbers are given in parentheses.

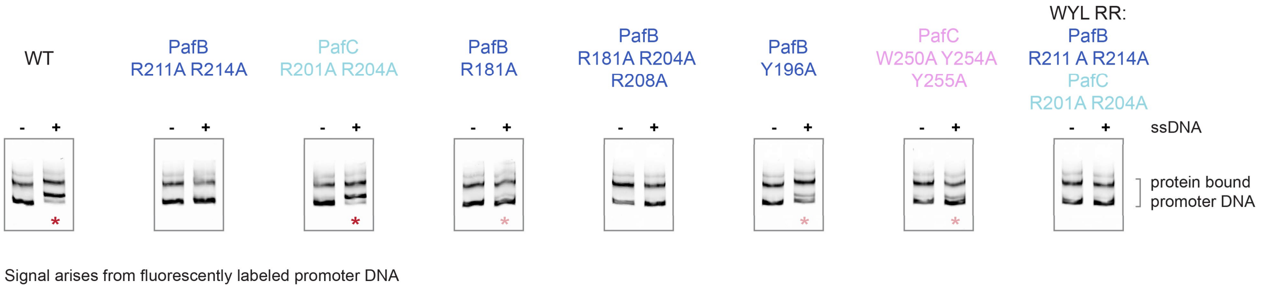

Fig. S9. Comparison of RNAP-PafBC complex formation between wild-type (WT) PafBC and PafBC variants. EMSAs were performed with fluorescently labeled promoter dsDNA containing the PafBC-specific promoter motif. All reactions included 0.2 µM promoter dsDNA, 0.5 µM RNAP, 1.5 µM SigA-RbpA, 1.5 µM CarD, and 3 µM PafBC. Reactions were compared in the presence and absence of 5 µM dT12 ssDNA. Asterisks mark a shift of the RNAP-σ^A band upon the addition of PafBC and ssDNA, red indicating a shift comparable to WT PafBC and pink indicating a shift to a lesser extent.

Table S1. Cryo-EM data collection parameters, model refinement and validation statistics.

**Data collection**		
	**Dataset 1**	**Dataset 2**
Detector	Gatan K3	Gatan K3
Acquisition mode	counting super-resolution	counting super-resolution
Nominal magnification	105	105
Voltage (kV)	300	300
Electron dose (e^-/Å)	81	76
Defocus range (µm)	-1.0 µm to -3.0 µm	-1.0 µm to -3.0 µm
Pixel size (Å)	0.84	0.84
	Particles of both datasets were pooled after 3D classification.	
Name of 3D reconstruction	**map$_{PafBC}$**	**map$_{RNAP}$**
EMDB map entry	EMD-50590	EMD-50589
PDB coordinate entry	PDB-9FND	-
Final number of particles images	154’302	470’245
Resolution (Å) (at FSC = 0.143)	4.0	3.3
	Both maps were combined to generate composite RNAP-PafBC map.	
Composite map		
EMDB map entry	EMD-50591	
PDB coordinate entry	PDB-9FNE	

**Refinement and validation statistics**		
	**PafBC**	**RNAP-PafBC**
Overall model geometry		
Clash score (all atoms)	6.54	5.11
MolProbity score	1.48	1.37
RMSD (bonds)	0.002	0.002
RMSD (angles)	0.423	0.417
Ramachandran plot (%)		
Favored	97.36	97.53
Allowed	2.64	2.47
Outliers	0.00	0.00
Rotamer outliers (%)	0.59	0.99
Cβ outliers (%)	0.00	0.00
Peptide plane (%)		
Cis proline / general	5.7/0.0	3.0/0.0
Twisted proline / general	0.0/0.0	0.0/0.0
CaBLAM outliers (%)	1.25	2.10
CC (mask)	0.74	0.74
CC (box)	0.59	0.64
Resolution estimates (Å)		
FSC (model vs. map, 0.5)	4.2	3.4

Table S2. Sequences of transcription assay templates and oligonucleotides used for EMSAs and cryo-EM sample preparation.

Within these sequences, the PafBC-specific promoter (composed of the -26 and the -10 motif) is highlighted in orange, the sequence of dimeric Broccoli is highlighted in light green, and the sequence of the associated tRNA scaffold is highlighted in red. Fluorescein labels are indicated as green FAM for labels at oligonucleotide ends, or as green T for Fluorescein-labeled thymines. Lowercase letters indicate non-complementary regions.

Purpose	Sequence (5' → 3')
**Transcription assay templates**	
PafBC-dependent promoter template (long)	CTGTCCGATCGGACCCGGGATGCTCCCACGGTAGGCCTGTCGTGTGCCGTCCG<br>GCGCAAAACCGCGATGCGGGTCCGGACAGCTCGGCGTGTCCACAGTATGGAGC<br>TAAGTGATGTGTTCCAGTGGTGAAGAGTTCGACCGGACTTGTCGGTGGTCTGC<br>TCTAACGTCACGGCCAACCGATCGGAACACCGGTCAGACACGACTACTCGGAG<br>AGGCACCACCATGGCGCAGCAGGCCCCAGATCGCGAAAAGGCCTGAGCCCGGA<br>TAGCTCAGTCGGTAGAGCAGCGGAGACGGTCGGGTCCATCTGAGACGGTCGGG<br>TCCAGATATTCGTATCTGTCGAGTAGAGTGTGGGCTCAGATGTCGAGTAGAGT<br>GTGGGCTCCGCGGGTCCAGGGTTCAAGTCCCTGTTCGGGCGCCA
PafBC-dependent promoter template (short)	CTGTCCGATCGGACCCGGGATGCTCCCACGGTAGGCCTGTCGTGTGCCGTCCG<br>GCGCAAAACCGCGATGCGGGTCCGGACAGCTCGGCGTGTCCACAGTATGGAGC<br>TAAGTGATGTGTTCCAGTGGTGAAGAGTTCGACCGGACTTGTCGGTGGTCTGC<br>TCTAACGTCACGGCCAACCGCCCGGATAGCTCAGTCGGTAGAGCAGCCCGGAT<br>AGCTCAGTCGGTAGAGCAGCGGAGACGGTCGGGTCCATCTGAGACGGTCGGGT<br>CCAGATATTCGTATCTGTCGAGTAGAGTGTGGGCTCAGATGTCGAGTAGAGTG<br>TGGGCTCCGCGGGTCCAGGGTTCAAGTCCCTGTTCGGGCGCCA
promoterless template	CTGTCCGATCGGACCCGGTTGATCGCCAAGGCTGTGGCCAACTCGTTGGCCAA<br>GAAAATGGCCGAGGTCCGCGGCGACGATGCCCACGAGGCGAAGTCGTACTTCC<br>TCAACATCAAGGGCCCCGAGCTGCTGAACAAATTCGTCGGGGAAACGGAACGC<br>CACATCCGGCTGATCTTCCAACGGGCCCGCGAGAAGGCGTCGGAAGGCACTCC<br>GGTGATCGTGATGGCGCAGCAGGCCCCAGATCGCGAAAAGGCCTGAGCCCGGA<br>TAGCTCAGTCGGTAGAGCAGCGGAGACGGTCGGGTCCATCTGAGACGGTCGGG<br>TCCAGATATTCGTATCTGTCGAGTAGAGTGTGGGCTCAGATGTCGAGTAGAGT<br>GTGGGCTCCGCGGGTCCAGGGTTCAAGTCCCTGTTCGGGCGCCA

primer for PCR amplification<br>*forward*	CTGTCCGATCGGACCCG
primer for PCR amplification<br>*reverse*	TGGCGCCCGAACAGG
**DNA templates for EMSAs investigating RNAP-PafBC complex formation**	
PafBC-specific promoter with transcription bubble<br>*forward strand*	**FAM**–TCGTCTACTGTGGTGAAGAGTTCGACCGGACTTGTCGGTGGTCTGCTCT<br>AACGTCACGGCCAACCGATCGGAACACC
PafBC-specific promoter with transcription bubble<br>*reverse strand*	GGTGTTCCGATCGGTaccggacatgtaaAGAGCAGACCACCGACAAGTCCGGT<br>CGAACTCTTCACCACAGTAGACGA
PafBC-specific promoter without transcription bubble<br>*forward strand*	**FAM**–TCGTCTACTGTGGTGAAGAGTTCGACCGGACTTGTCGGTGGTCTGCTCT<br>AACGTCACGGCCAACCGATCGGAACACC
PafBC-specific promoter without transcription bubble<br>*reverse strand*	GGTGTTCCGATCGGTTGGCCGTGACGTTAGAGCAGACCACCGACAAGTCCGGT<br>CGAACTCTTCACCACAGTAGACGA
housekeeping promoter with transcription bubble<br>*forward strand*	CCCCGTTTTGGGGCAgatgcgattctgaAATACTGCTTTGTCTGGCTTGTCAA<br>GTCGCTCTGGACCGGGTTGTTTTC
housekeeping promoter with transcription bubble<br>*reverse strand*	**FAM**–GAAAACAACCCGGTCCAGAGCGACTTGACAAGCCAGACAAAGCAGTATT<br>AAGCTGGCAGGGTTGCCCCAAAACGGGG
nonspecific DNA with transcription bubble<br>*forward strand*	**FAM**–GTCGTGAAGGACACCGCCGAAGCGCCCAAGACGGCCGACGAGAAAGCCG<br>CCGAGGCCACCGGGCCCAGCAAGGTCAA

nonspecific DNA with transcription bubble *reverse strand*	TTGACCTTGCTGGGCgtcagcatgcgaaCGGCTTTCTCGTCGGCCGTCTTGGG CGCTTCGGCGGTGTCCTTCACGAC
**Oligonucleotides used for cryo-EM sample preparation**	
PafBC-specific promoter with transcription bubble *forward strand*	GTGGTGAAGAGTTCGACCGGACTTGTCGGTGGTCTGCTCTAACGTCACGGCCA ACCGATCGGAACACC
PafBC-specific promoter with transcription bubble *reverse strand*	GGTGTTCCGATCGGTaccggacatgtaaAGAGCAGACCACCGACAAGTCCGGT CGAACTCTTCACCAC
ssDNA	TTGTTGTTGTT
**Oligonucleotides for EMSAs investigating PafBC binding to ssDNA without free ends**	
blocked end ssDNA	GGGCCCGGGCCCGGTA**T**ATCCGGGCCCGGGCCCTTTTTTTTTTTCCCGGGCC CGGGCCTATATGGCCCGGGCCCGGG
hairpin DNA	GGGCCCGGGCCCGGTA**T**ATCCGGGCCCGGGCCC
